# Supplementary material for: Homolytic H2 dissociation for enhanced hydrogenation catalysis on oxides
Source: Nat Commun. 2024 Jan 15;15:540. doi: 10.1038/s41467-024-44711-7 (PMC10789776; doi:10.1038/s41467-024-44711-7)
Supplement: Supplementary file 1 — Supplementary information [file 41467_2024_44711_MOESM1_ESM.pdf]

Supplementary Information

Homolytic H_2 dissociation for enhanced hydrogenation catalysis on oxides

Chengsheng Yang[1], Sicong Ma*[,2], Yongmei Liu[1], Lihua Wang[3], Desheng Yuan[1], Wei-Peng Shao[4], Lunjia Zhang[4], Fan Yang[4], Tiejun Lin[5], Hongxin Ding [1], Heyong He [1], Zhi-Pan Liu[1,2], Yong Cao[1], Yifeng Zhu*[,1], Xinhe Bao*[,1,6]

[1]Department of Chemistry, Shanghai Key Laboratory of Molecular Catalysis and Innovative Materials, Collaborative Innovation Center of Chemistry for Energy Materials, Fudan University, Shanghai 200438

[2]Key Laboratory of Synthetic and Self-Assembly Chemistry for Organic Functional Molecules, Shanghai Institute of Organic Chemistry, Chinese Academy of Sciences, Shanghai 200032

[3]Shanghai Synchrotron Radiation Facility, Shanghai Advanced Research Institute, Chinese Academy of Sciences, Shanghai 201204

[4]School of Physical Science and Technology, Shanghai Tech University, Shanghai 201210

[5]Key Laboratory of Low-Carbon Conversion Science and Engineering, Shanghai Advanced Research Institute, Chinese Academy of Sciences, Shanghai 201210

[6]State Key Laboratory of Catalysis, National Laboratory for Clean Energy, Collaborative Innovation Center of Chemistry for Energy Materials, Dalian Institute of Chemical Physics, Chinese Academy of Sciences, Dalian 116023

*Corresponding Authors:

zhuyifeng@fudan.edu.cn;

scma@mail.sioc.ac.cn;

xhbao@dicp.ac.cn

Contents

Supplementary Methods

Supplementary Figures 1-56

Supplementary Tables 1-11

Supplementary References

Supplementary Methods

Synthesis of Cu/Ga_2O_3 and Cu/SiO_2 catalysts:

Cu/Ga_2O_3 and Cu/SiO_2 catalysts were prepared by equal volume impregnation method with ca. 5wt% loading amount of Cu. Specifically, 0.32 g of $Cu(NO_3)_2{\cdot}xH_2O$ was dissolved in 2 mL of deionized water to form a transparent solution. Then, the transparent solution was added dropwise into 2 g of Ga_2O_3 with different crystal phase or SiO_2 powder. After ultrasonic treatment for 3 h, the semi-liquid solid was dried at 333 K for 12 h and further calcined at 723 K for 3 h with a ramp rate of 2 K/min, and the obtained samples are labeled as Cu/Ga_2O_3 and Cu/SiO_2, respectively.

Supplementary note 1: Calculation of number of surface Ga-H and -OH via H_2-exchange (titration).

Transient kinetic analysis with mass spectroscopy (TKA-MS) is performed via **titration** through a sample loop. The coverage of surface Ga-H and -OH were calculated based on the assumption that the HD (*m/z*=3) came from the H_2 reacted with Ga-D and Ga-OD, respectively. The as-formed surface Ga-OD would be exchanged with H_2 at 150 °C (Ga-OD + H_2 → Ga-OH + HD), while the as-formed surface Ga-D would be exchanged with H_2 forming HD with *m/z* of 3 (Ga-D + H_2→ Ga-H + HD) at higher temperature of 350 °C. Thus, the amount of Ga-D or Ga-OD can be distinguished by the different H-D exchange temperature, and the amount of surface Ga-D or Ga-OD is equal to the H_2 consumption. Specifically, the amount of surface Ga-D is equal to the H_2 consumption at 350 °C in H_2-exchange experiment. The amount of surface Ga-OD is equal to the H_2 consumption at 150 °C in H_2-exchange experiment. The H_2 consumption was calculated by the area of inverted peak in H_2-exchange experiment, as shown in equation:

$$\Delta F\ (H2)(\mu L) = \frac{\sum(A\ (Stable) - Ai)}{A(Stable)} \times 200\mu L \quad (1)$$

$$Consumption_{H2}(mmol\ g^{-1}) = \frac{\Delta F\ (H2)/1000}{22.4\text{mL/mmol} \times m_{\text{catalyst}}} \quad (2)$$

where $\Delta F\ (H2)$ is the H_2 consumption in H_2-exchange experiment. $A\ (Stable)$ is the inverted peak area of H_2 when the area reached stable. Ai is the inverted peak area of H_2 before the area reached stable. m_{catalyst} is the mass of the catalyst. The difference between $A\ (Stable)$ and Ai represents the consumption of hydrogen participating in the exchange reaction.

Supplementary note 2: Calculation of surface exposed Ga^{3+} density

The S_{Ga2O3}, V_{Ga2O3}, and ρ_{Ga2O3} are the surface area, volume density and crystal density of Ga_2O_3 nanoparticle with the diameter of d respectively. Their calculation equation are as follows:

$$S_{\text{Ga2O3}}\ (nm^2) = \pi d^2 \quad (3)$$

$$V_{\text{Ga2O3}}\ (nm^3) = \frac{\pi d^3}{6} \quad (4)$$

$$\rho_{\mathrm{Ga2O3}}(g\ cm^{-3}) = \frac{Z \times M}{V \times N_A} \tag{5}$$

$$Theoretical\ area\ (m^2\ g^{-1}) = \frac{S_{\mathrm{Ga2O3}}}{V_{\mathrm{Ga2O3}} \times \rho_{\mathrm{Ga2O3}}} \tag{6}$$

where Z is the number of Ga_2O_3 molecules in the unit cell. M is molecular weight of Ga_2O_3. V is unit cell volume. N_A is Avogadro constant.

$\rho_{\mathrm{surface\ Ga}}$ is the average Ga atomic density on each crystal plane:

$$\rho_{surface\ Ga}(atoms\ nm^{-2}) = \frac{number\ of\ Ga\ atoms}{area\ of\ crystal\ plane} \tag{7}$$

Based on the crystal phase of Ga_2O_3, the average Ga atomic density of the low-index surface ([100], [010], [001], [110], [101], [011] and [111]) were summarized in **Supplementary Table 3**, the average Ga atomic density on Ga_2O_3 surface is obtained.

$$c_{surface\ Ga}(mmol\ g^{-1}) = \frac{S_{\mathrm{Ga2O3}} \times \rho_{surface\ Ga}/N_A}{\rho_{\mathrm{Ga2O3}} \times V_{\mathrm{Ga2O3}}} \tag{8}$$

Supplementary Figures

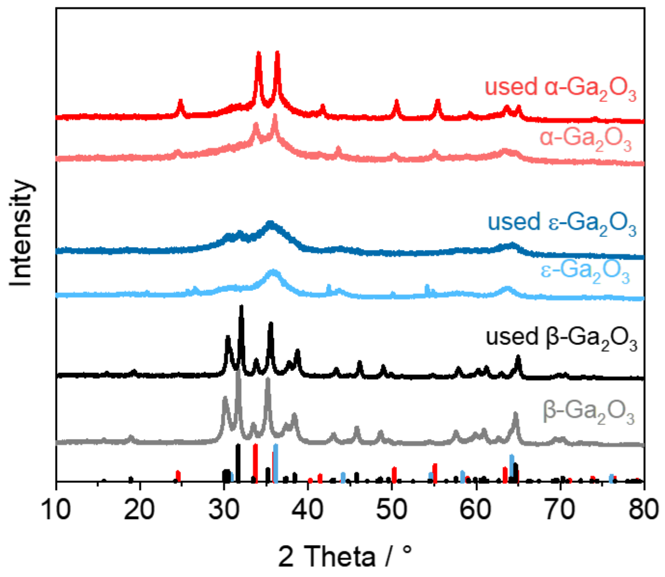

Supplementary Figure 1. XRD pattern of fresh and used Ga_2O_3 samples.

Note: XRD of both fresh and used α-Ga_2O_3 displayed diffraction peaks at 2 theta of 24.5°, 36.0°, and 55.1°, which are indexed to the (011), (110) and (116) crystal facets of the hexagonal α-Ga_2O_3 (Red vertical lines, JCPDS NO. 06-0503).[1] Accordingly the broad and weak XRD peaks indicated the existence of the poorly ordered ε-Ga_2O_3 crystal phase. The diffraction peaks at 30.8°, 36.2°, and 64.2° are attributed to the (220), (311), and (440) crystal facets of the cubic ε-Ga_2O_3 (Blue vertical lines, JCPDS NO. 20-0426).[2] XRD of β-Ga_2O_3 displayed diffraction peaks of 31.7°, 35.2°, and 64.7°, which are indexed to the (-202), (111), and (-712) crystal facets of the monoclinic β-Ga_2O_3 (Black vertical lines, JCPDS NO. 41-1103).[1]

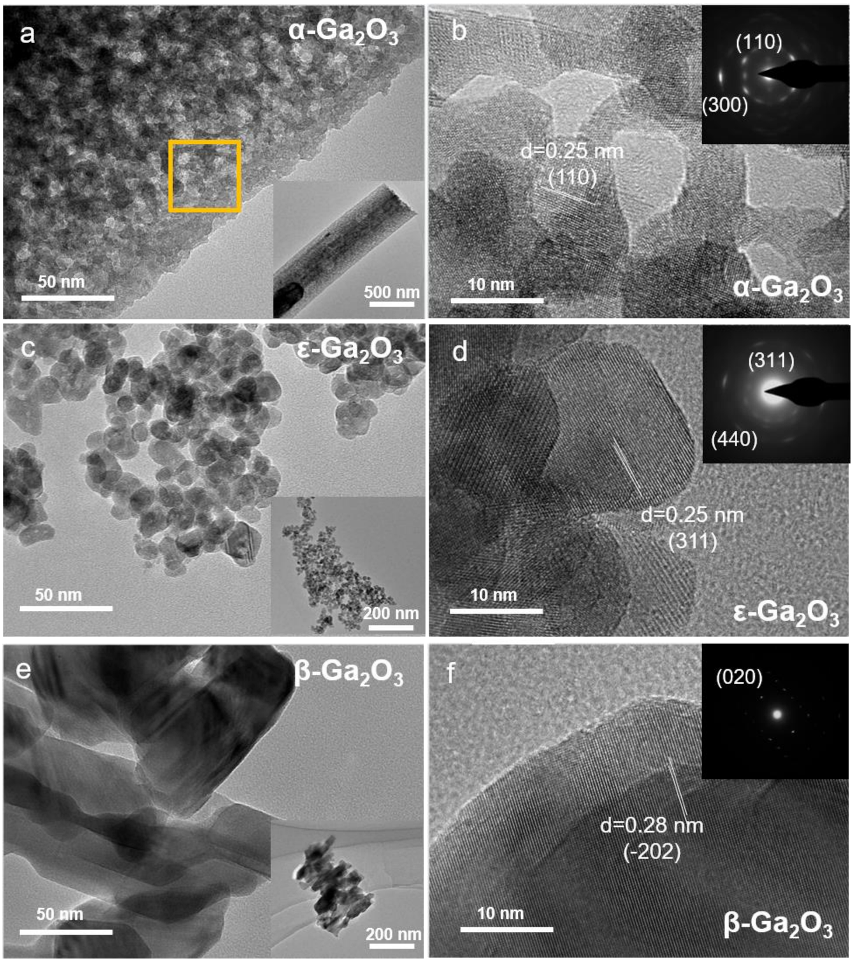

Supplementary Figure 2. Morphography of different Ga_2O_3. **a** HRTEM side view images of α-Ga_2O_3. **b** Enlarged image of a selected area with orange frame in a, and the inset showing the selected area electron diffraction patterns of α-Ga_2O_3. **c** HRTEM side view images of ε-Ga_2O_3. **d** Enlarged image and the inset showing the selected area electron diffraction patterns of ε-Ga_2O_3. **e** HRTEM side view images of β-Ga_2O_3. **f** Enlarged image and the inset showing the selected area electron diffraction patterns of β-Ga_2O_3.

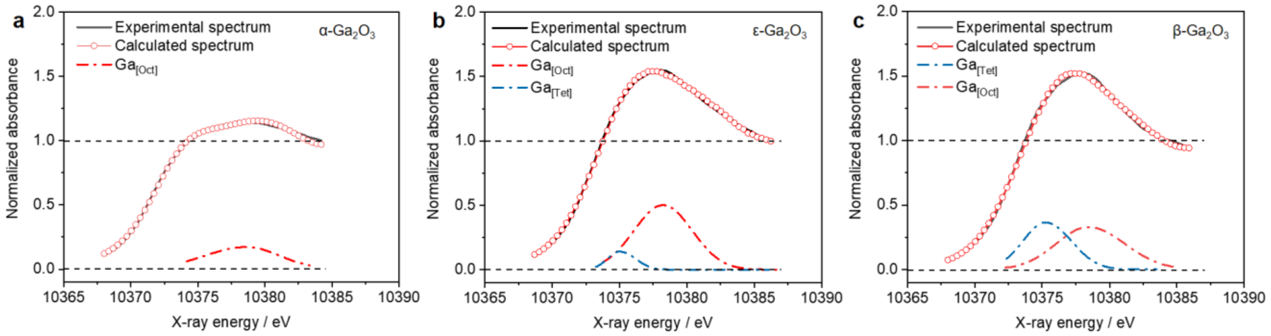

Supplementary Figure 3. Ga K-edge XANES of Ga_2O_3 (black solid line) and the simulated spectrum (red circle), which is composed of two kinds of Ga^{3+} ($Ga_{[Oct]}$, $Ga_{[Tet]}$). α-Ga_2O_3 (a), ε-Ga_2O_3 (b) and β-Ga_2O_3 (c).

Note：The deconvolution analysis of XANES spectra was carried out following the method in the previous study to estimate the ratio of $Ga_{[Oct]}$/$Ga_{[Tet]}$ quantitatively.[3,4] Supplementary Fig. 3 illustrates deconvoluted spectra of Ga_2O_3. We assumed that each component consists of an arctangent curve for continuum absorption and a Gaussian curve for the white line. The positions of two Gaussian peaks were determined to be 10375 and 10379 eV, which were identical to those of $Ga_{[Tet]}$ and $Ga_{[Oct]}$, respectively.

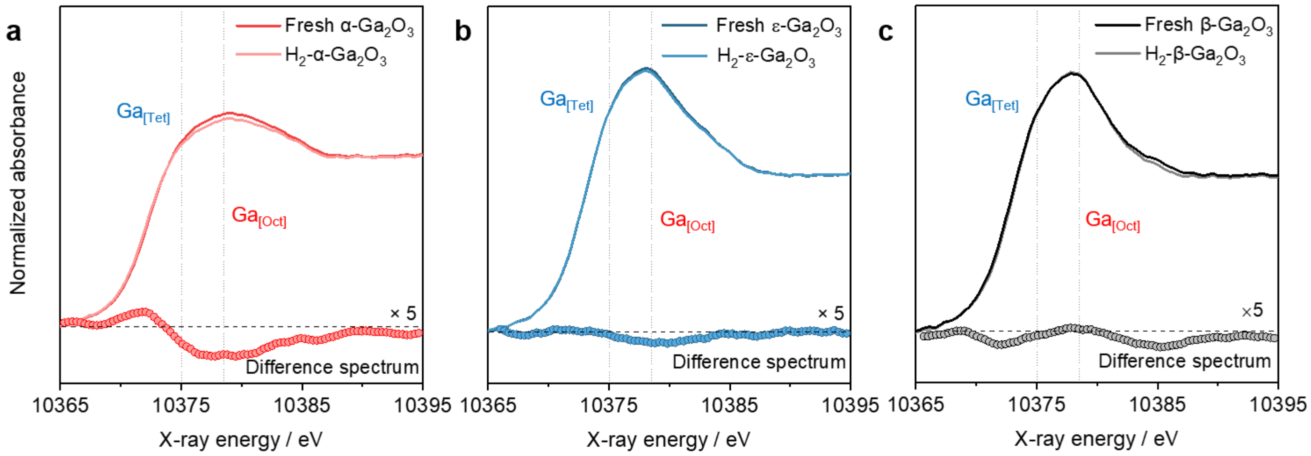

Supplementary Figure 4. Ga K-edge XANES of fresh Ga_2O_3 and Ga_2O_3 samples contacting with H_2 for 15 min at 350 °C, 1 MPa. α-Ga_2O_3 (a), ε-Ga_2O_3 (b) and β-Ga_2O_3 (c).

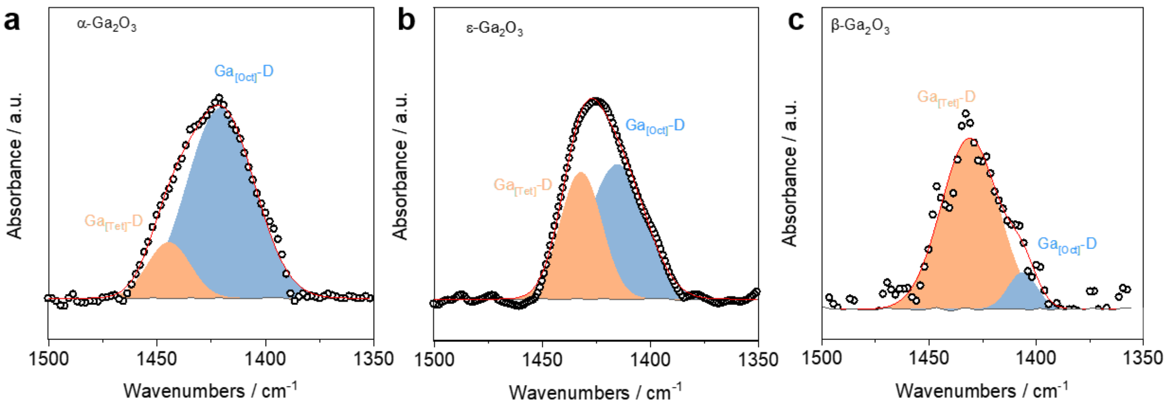

Supplementary Figure 5. D_2-IR for α-Ga_2O_3 (a), ε-Ga_2O_3 (b) and β-Ga_2O_3 (c) samples contacting with D_2 at 350 °C, 1 MPa.

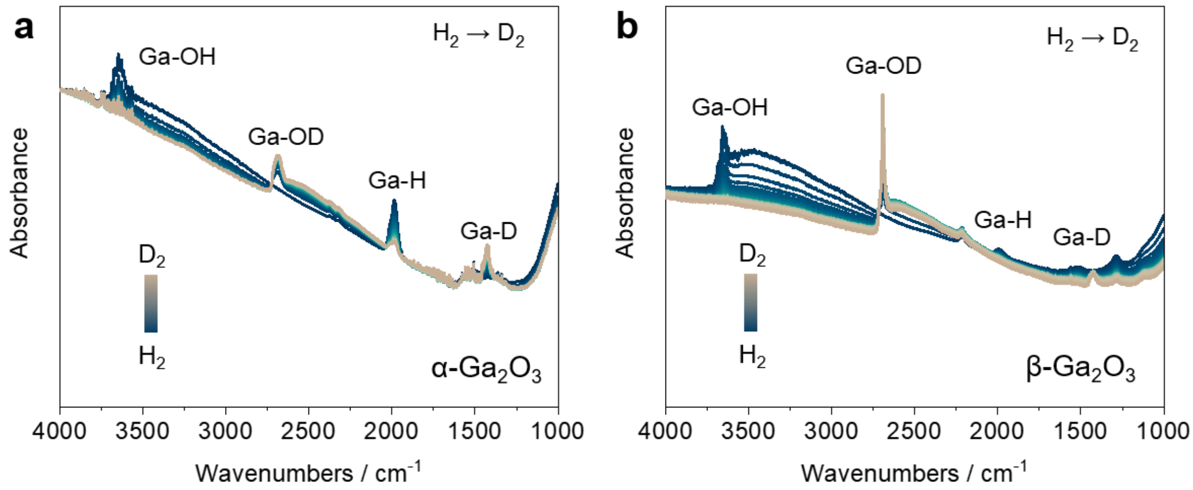

Supplementary Figure 6. a-b *In-situ* FTIR of α-Ga_2O_3 samples contacting H_2 to D_2 at 350 °C, 1 MPa. **c-d** *In-situ* FTIR of β-Ga_2O_3 samples contact H_2 to D_2 at 350 °C, 1 MPa.

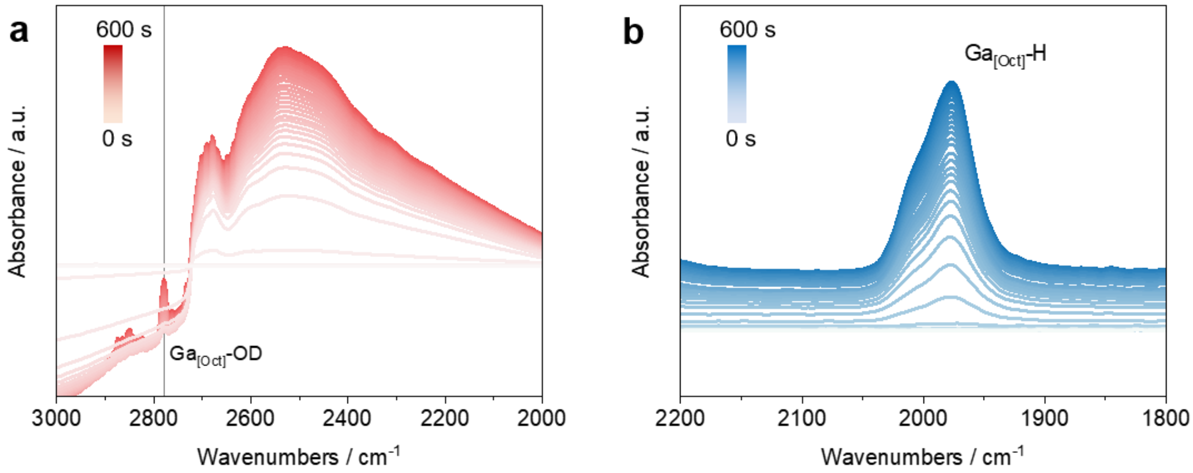

Supplementary Figure 7. **a** D_2-IR of α-Ga_2O_3 samples contacting with D_2 at 350 °C, 1 MPa. **b** H_2-IR of α-Ga_2O_3 samples contacting with H_2 at 350 °C, 1 MPa. H_2 or D_2 was inlet into cell at 0 s.

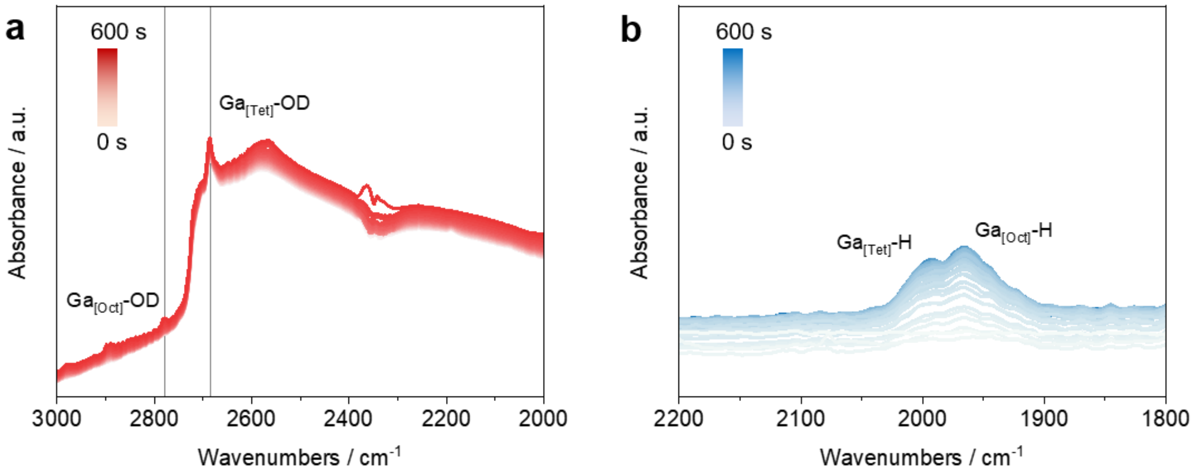

Supplementary Figure 8. a D_2-IR of ε-Ga_2O_3 samples contacting with D_2 at 350 °C, 1 MPa**. b** H_2-IR of ε-Ga_2O_3 samples contacting with H_2 at 350 °C, 1 MPa. H_2 or D_2 was inlet into cell at 0 s.

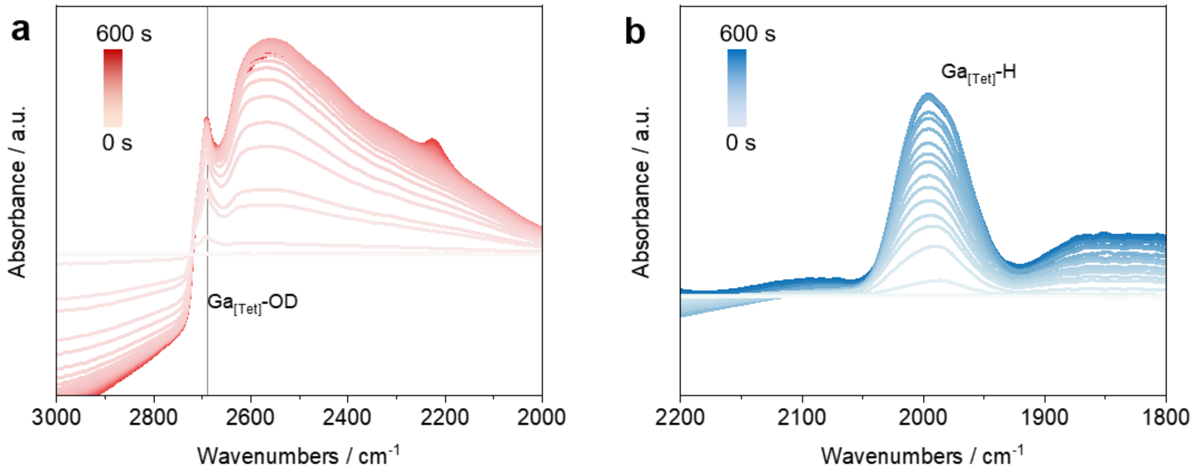

Supplementary Figure 9. **a** D_2-IR of β-Ga_2O_3 samples contacting with D_2 at 350 °C, 1 MPa. **b** H_2-IR of β-Ga_2O_3 samples contacting with H_2 at 350 °C, 1 MPa. H_2 or D_2 was inlet into cell at 0 s.

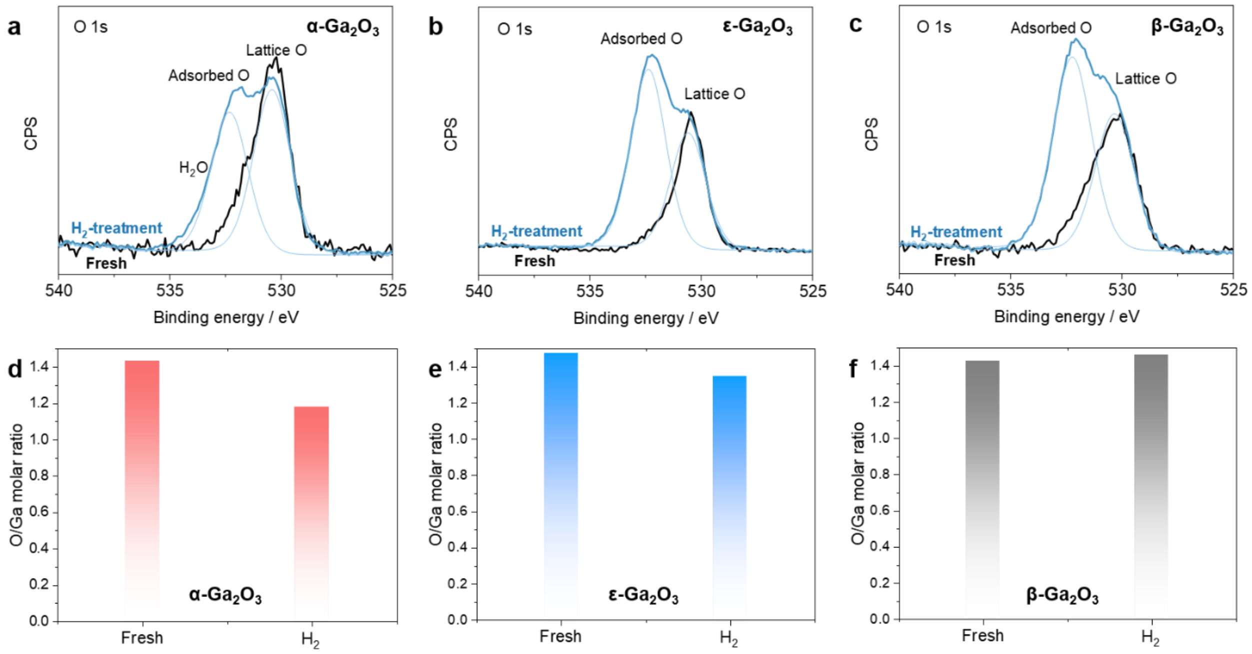

Supplementary Figure 10. a-c *In-situ* XPS results of O 1s for fresh and reduced Ga_2O_3. **d-f** O/Ga molar ratio of fresh and reduced Ga_2O_3.

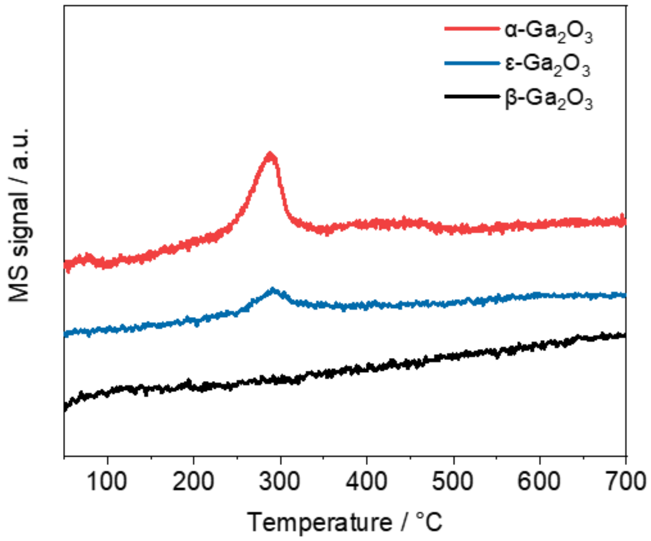

Supplementary Figure 11. TPR of different Ga_2O_3 samples (MS signal, *m*/*z*=18).

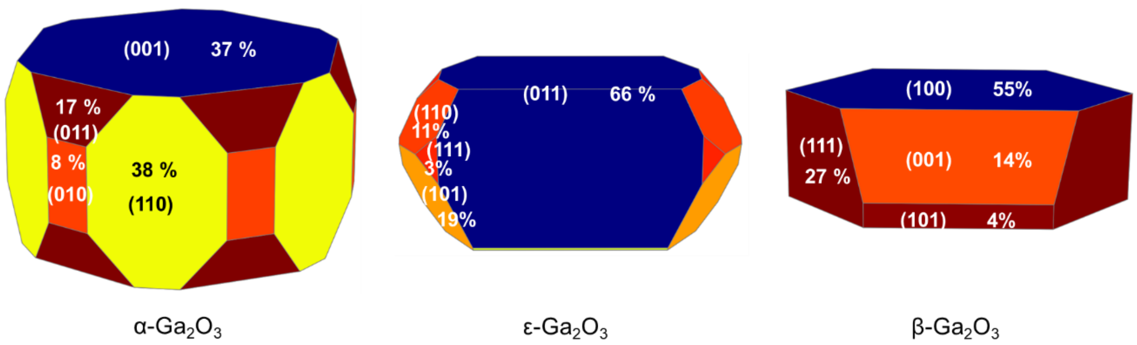

Supplementary Figure 12. The thermodynamic Wulff morphology of three Ga_2O_3 crystals.

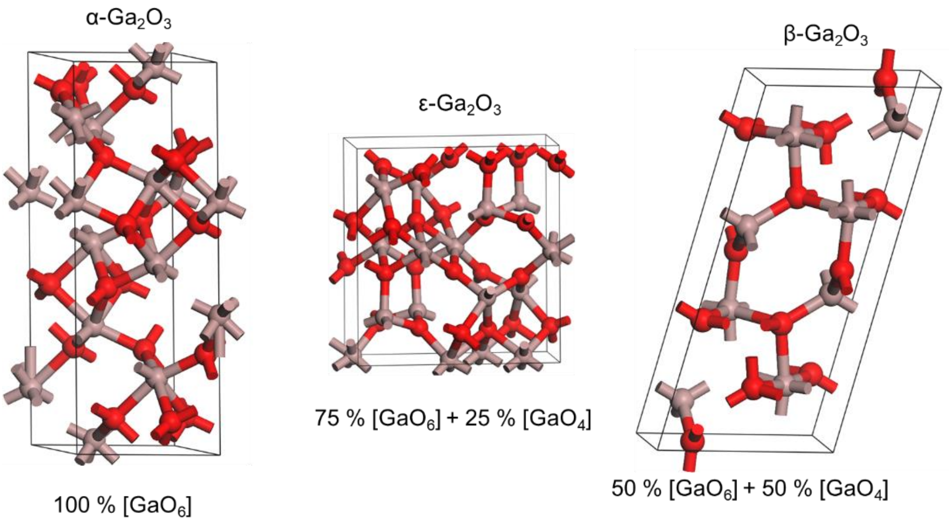

Supplementary Figure 13. The models of bulk α-Ga_2O_3, bulk ε-Ga_2O_3 and bulk β-Ga_2O_3.

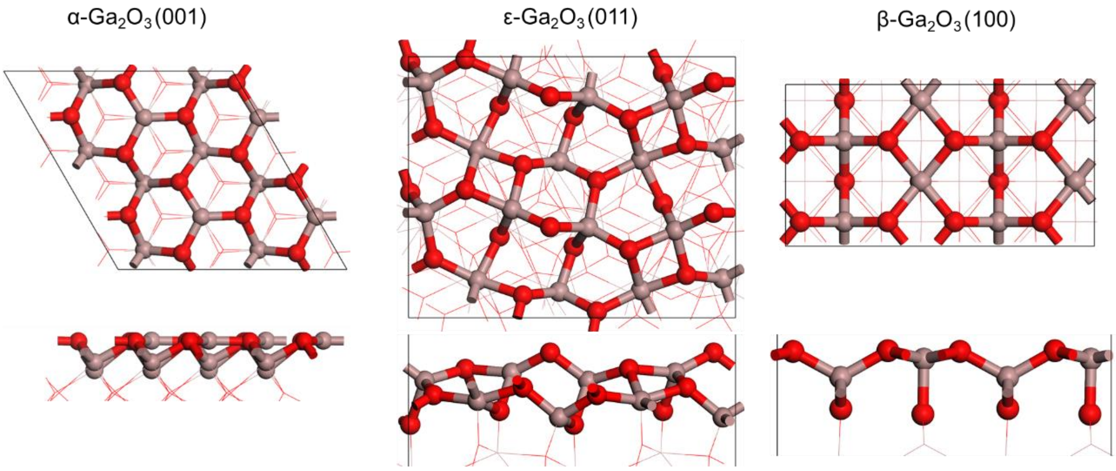

Supplementary Figure 14. The models of α-Ga_2O_3(001) surface, ε-Ga_2O_3(011) surface and β-Ga_2O_3(100) surface.

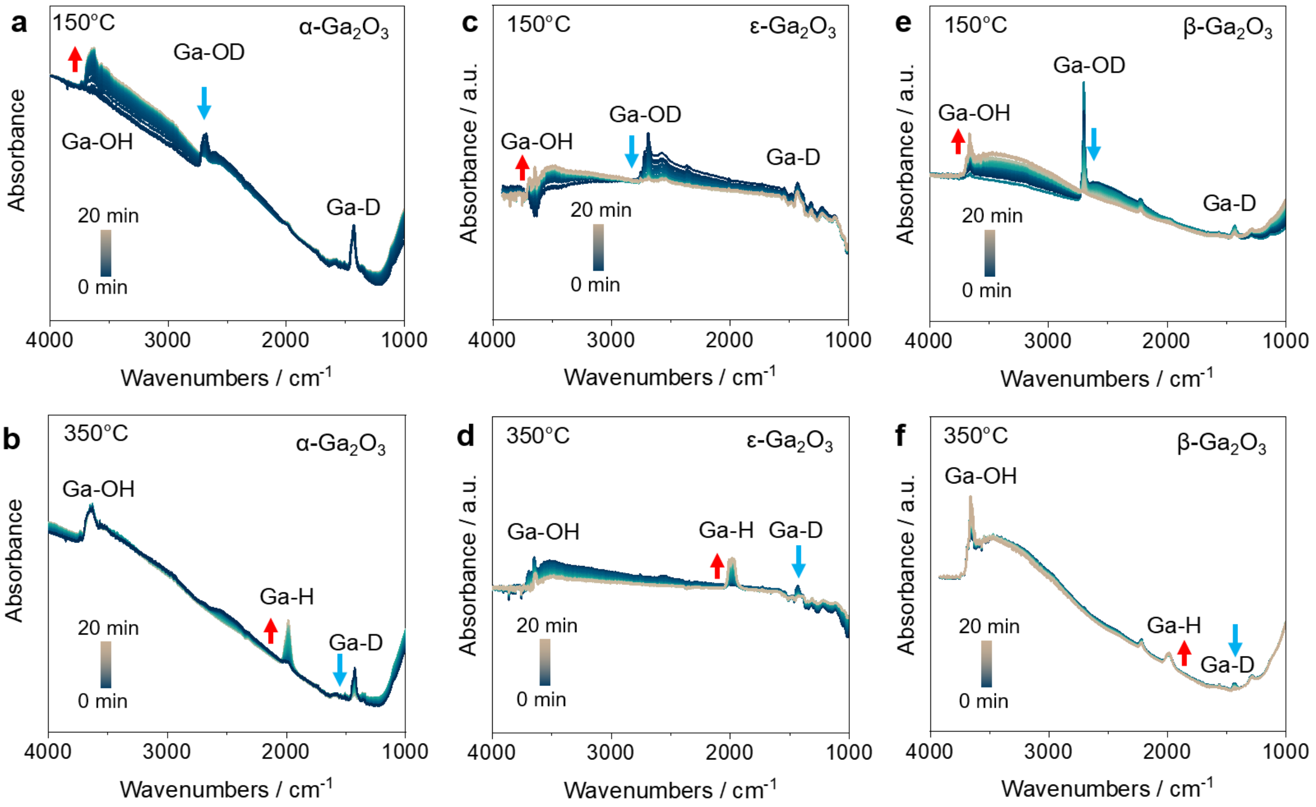

Supplementary Figure 15. The full IR spectrum with blank transmission cell as the background. **a** IR spectra of α-Ga_2O_3 during H_2-exchange at 150 °C after the catalysts were saturated with D_2 at 350 °C. **b** IR spectra of α-Ga_2O_3 during H_2-exchange at 350 °C after the catalysts were saturated with D_2 at 350 °C. **c** IR spectra of ε-Ga_2O_3 during H_2-exchange at 150 °C after the catalysts were saturated with D_2 at 350 °C. **d** IR spectra of ε-Ga_2O_3 during H_2-exchange at 350 °C after the catalysts were saturated with D_2 at 350 °C. **e** IR spectra of β-Ga_2O_3 during H_2-exchange at 150 °C after the catalysts were saturated with D_2 at 350 °C. **f** IR spectra of β-Ga_2O_3 during H_2-exchange at 350 °C after the catalysts were saturated with D_2 at 350 °C.

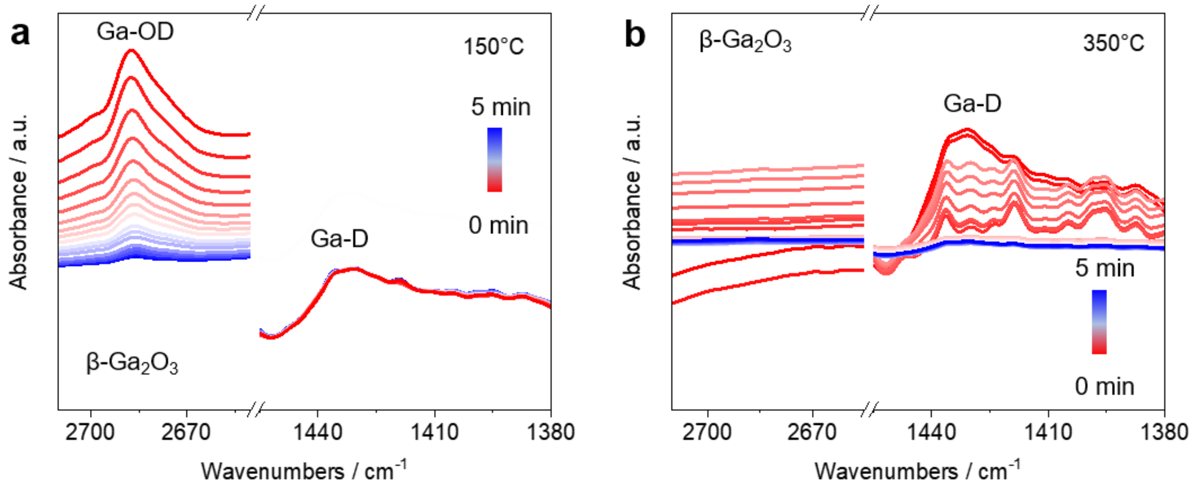

Supplementary Figure 16. FTIR spectra in TKA-IR experiments. **a** The IR spectra of β-Ga_2O_3 during H_2-exchange experiment at 150 °C after the catalysts were saturated with D_2 at 350 °C. **b** The IR spectra of β-Ga_2O_3 during H_2-exchange experiment at 350 °C after the catalysts were saturated with D_2 at 350 °C.

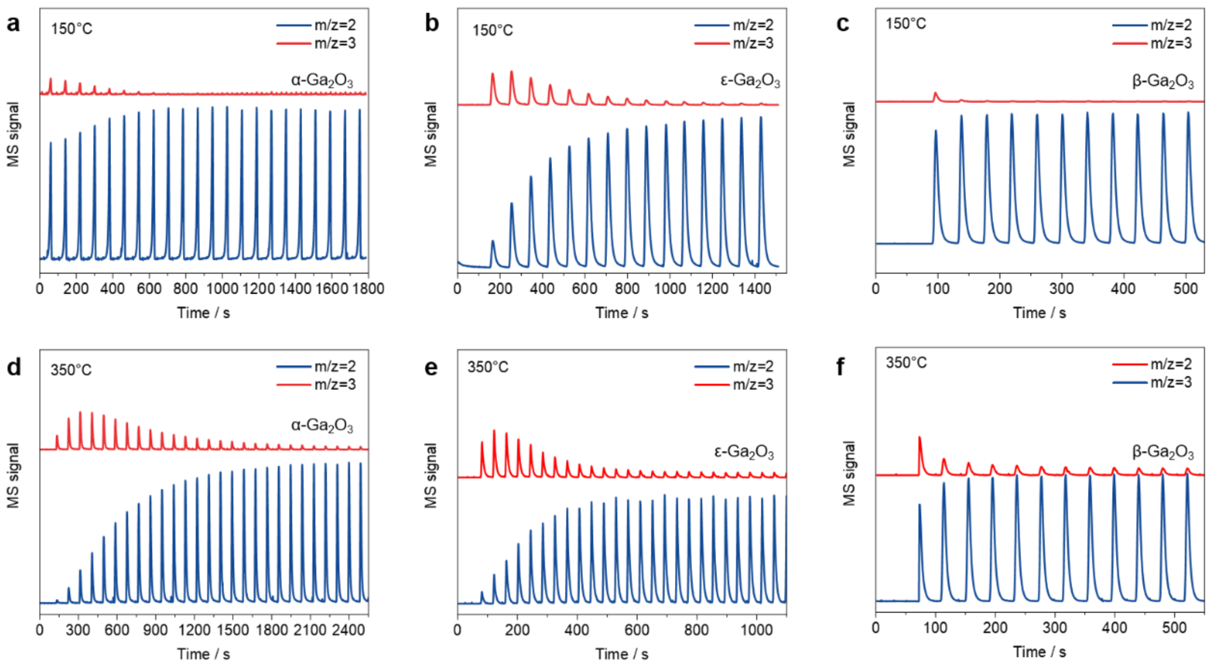

Supplementary Figure 17. MS signal in TKA-MS experiments. **a-c** Hydrogen consumption with *m/z*=2 (H_2), *m/z*=3 (HD) of Ga_2O_3 during H_2-exchange experiment at 150 °C after the catalysts were saturated with D_2 at 350 °C. **d-f** Hydrogen consumption with *m/z*=2 (H_2), *m/z*=3 (HD) of Ga_2O_3 during H_2-exchange experiment at 350 °C after the catalysts were saturated with D_2 at 350 °C.

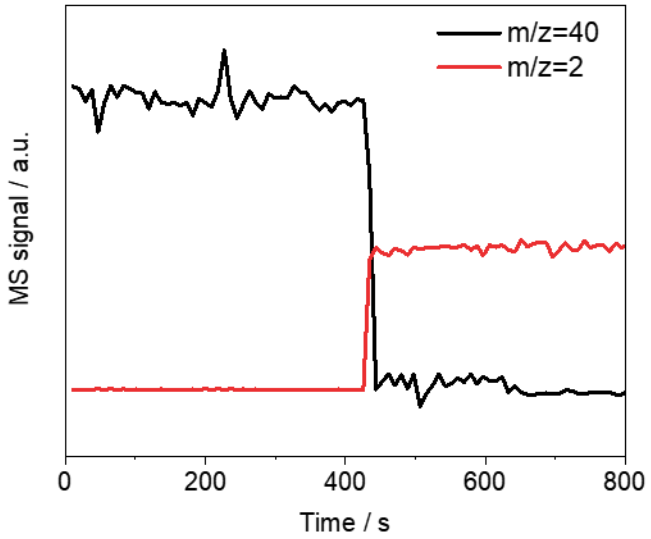

Supplementary Figure 18. The MS signal when Ar flow is replaced by H_2 flow in H_2-IR experiment.

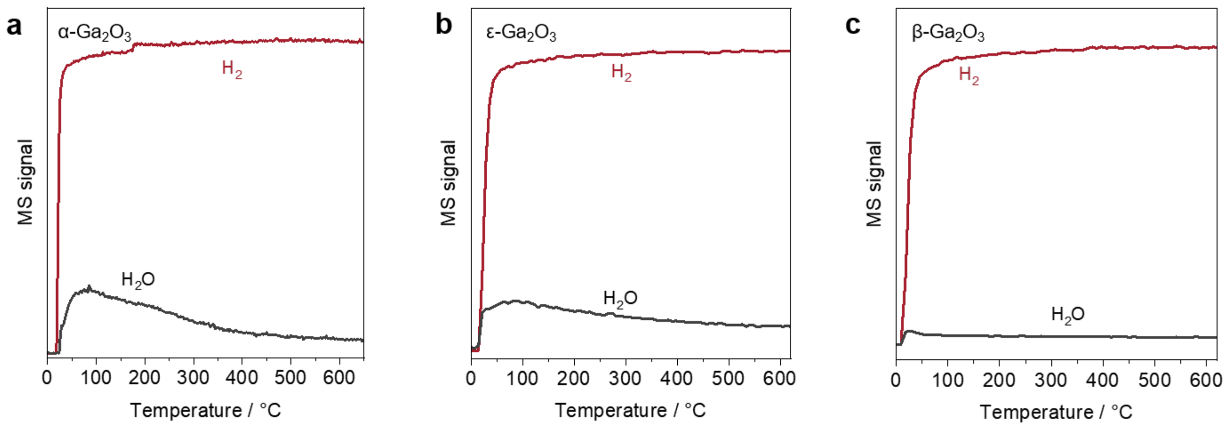

Supplementary Figure 19. The generation of H_2O (*m/z*=18) over α-Ga_2O_3 (a), ε-Ga_2O_3 (b) and β-Ga_2O_3 (c) samples with contact time of H_2 at 350 °C, 1 MPa.

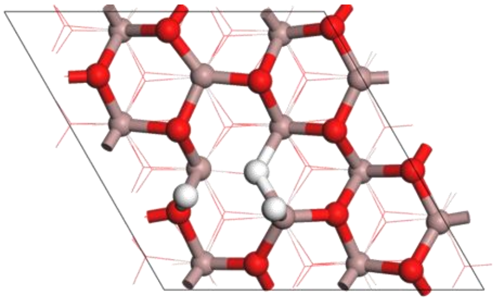

Supplementary Figure 20. H_2 adsorption energy by homolytic dissociation over hydrogenated and O-defective α-Ga_2O_3 (001). Red ball: O atom, grey ball: Ga atom, white ball: H atom.

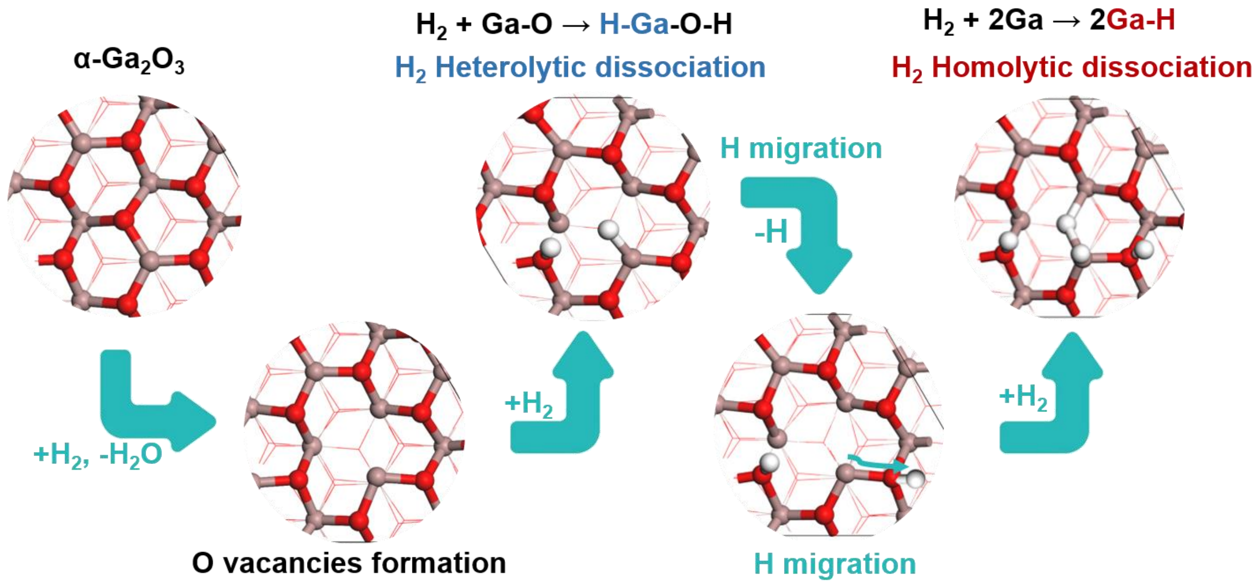

Supplementary Figure 21. Scheme of the homolytic dissociation and the migration of H over α-Ga_2O_3.

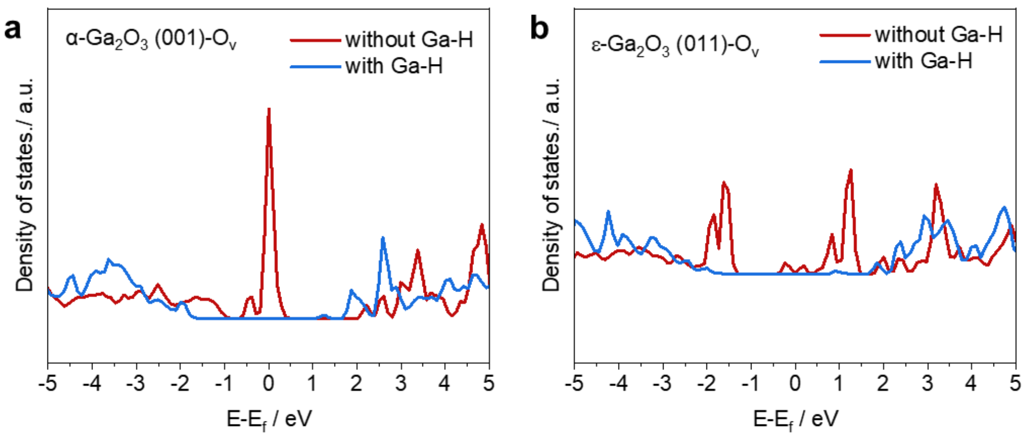

Supplementary Figure 22. The density of states of Ga 4p orbital for Ga_2O_3. **a** The density of states of Ga 4p orbital for the O-defective α-Ga_2O_3 (001) surfaces with/without the formation of GaH species. **b** The density of states of Ga 4p orbital for the O-defective ε-Ga_2O_3 (011) surfaces with/without the formation of GaH species.

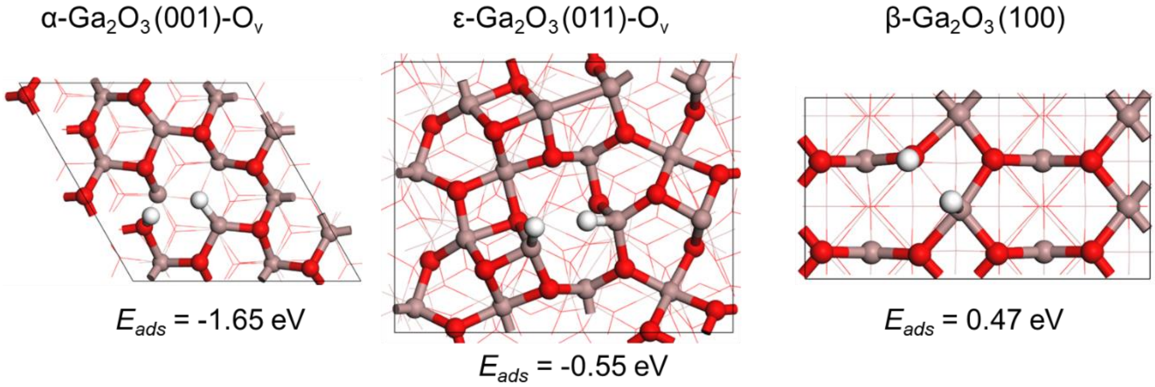

Supplementary Figure 23. The models of OH and Ga-H site on α-Ga_2O_3(001) surface with oxygen vacancy (O_v), ε-Ga_2O_3(011) surface with oxygen vacancy (O_v) and β-Ga_2O_3(100) surface.

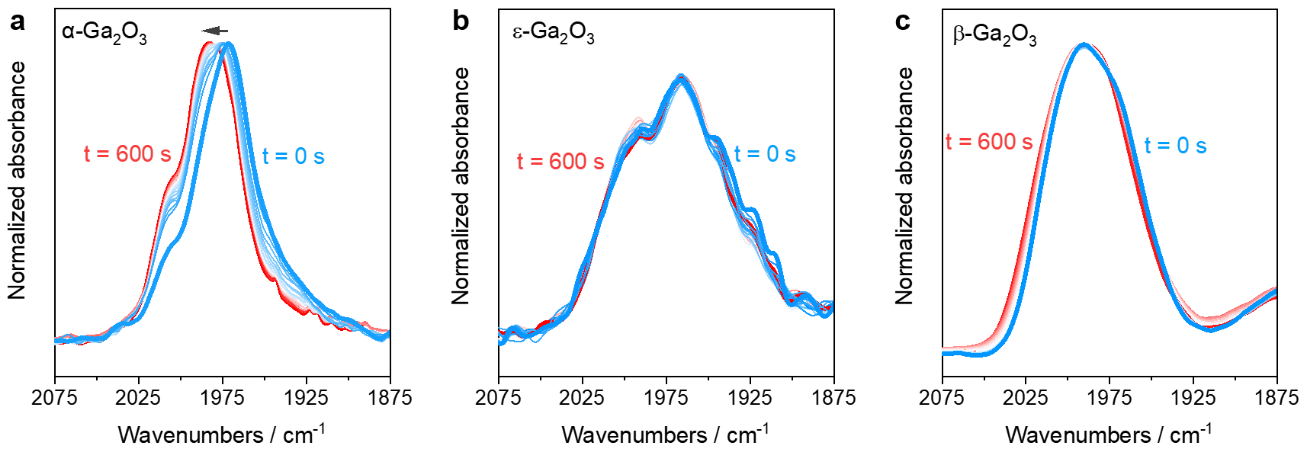

Supplementary Figure 24. Evolution of Ga-H band position of α-Ga_2O_3 (a), ε-Ga_2O_3 (b) and β-Ga_2O_3 (c) samples during H_2 treatment. The Ga-H peaks were normalized in intensity for better comparison.

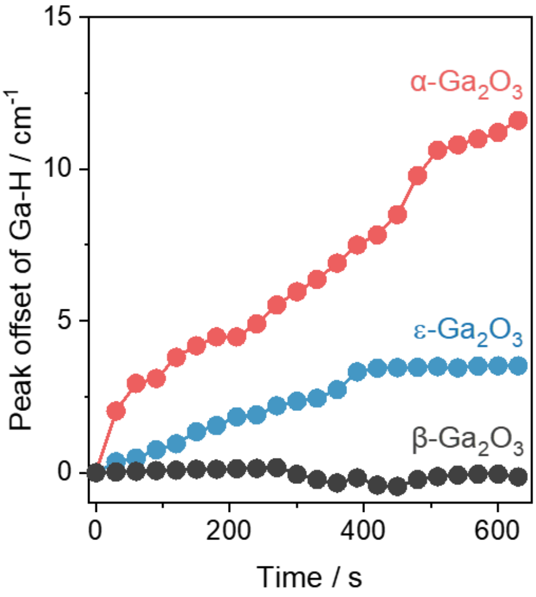

Supplementary Figure 25. IR characteristic peak offsets of Ga-H over Ga_2O_3 samples during H_2 treatment.

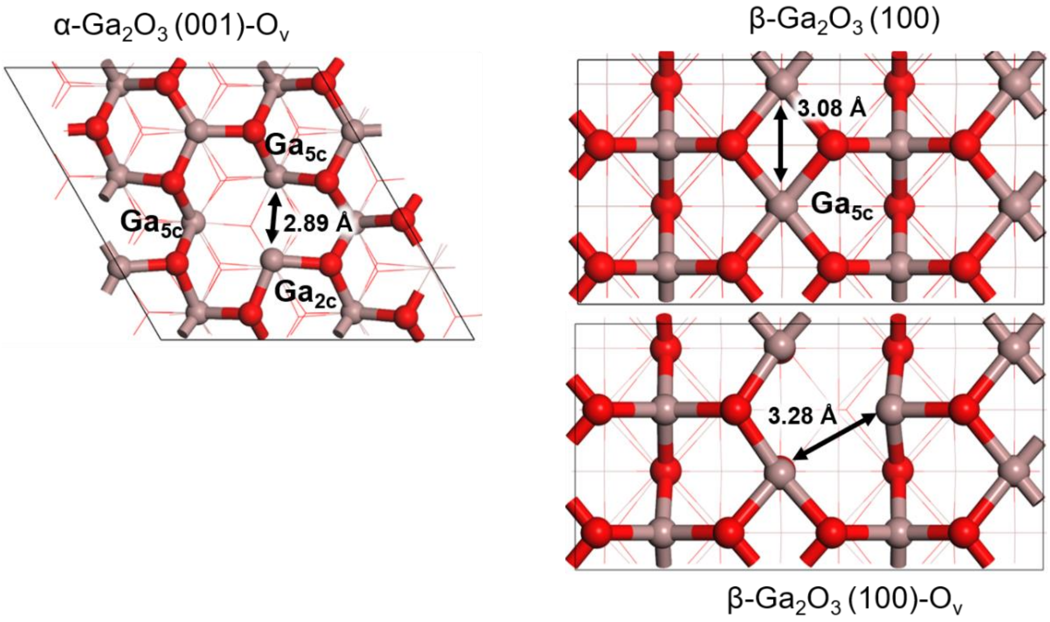

Supplementary Figure 26. The models and distance of Ga-Ga site on α-Ga_2O_3 (001) surface with oxygen vacancy (O_v) and β-Ga_2O_3 (100) surface.

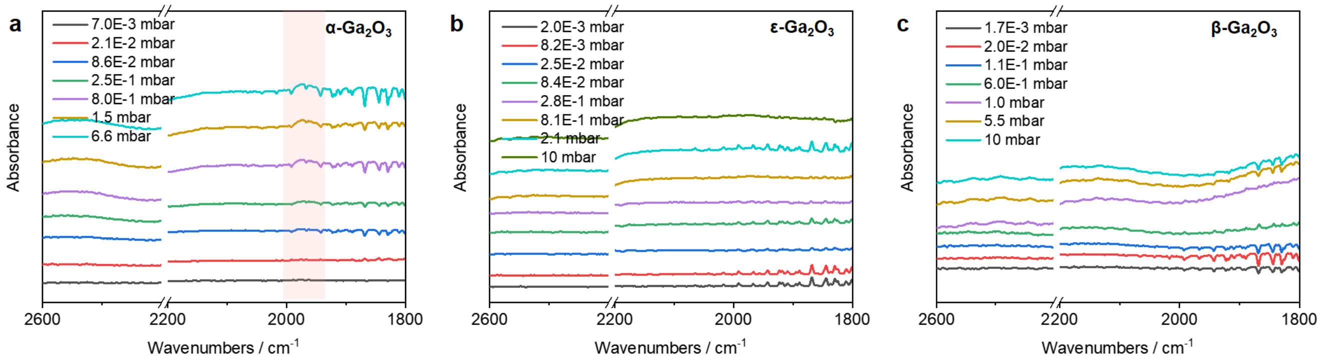

Supplementary Figure 27. H_2-chemisorption intensity of α-Ga_2O_3 (a), ε-Ga_2O_3 (b) and β-Ga_2O_3 (c) samples under different H_2 partial pressure measured by IR.

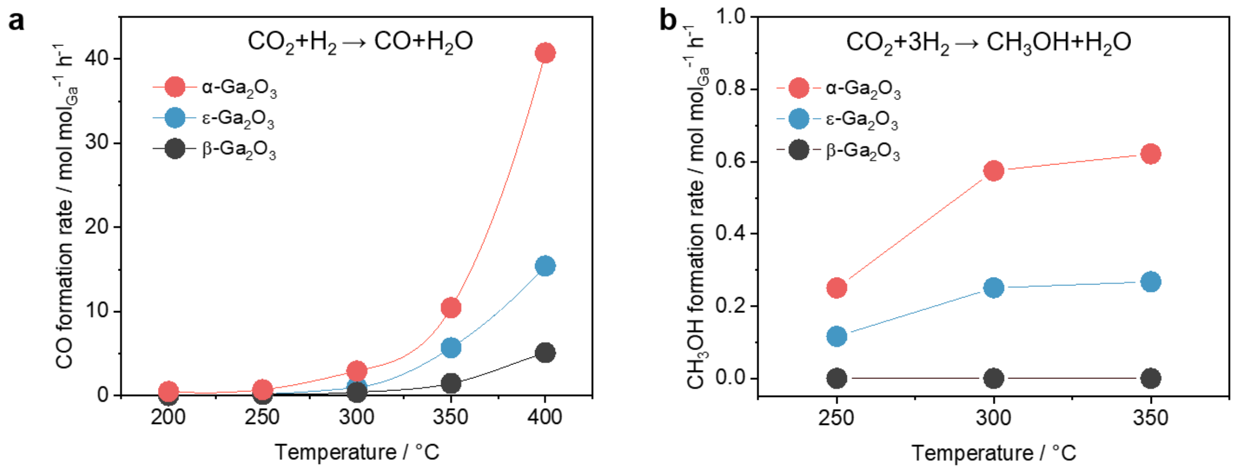

Supplementary Figure 28. **a** CO formation rate over Ga_2O_3 during rWGS. Reaction conditions: H_2/CO_2= 3 (v/v), 200-400 °C, 0.2 MPa, 9000 mL/g/h. **b** CH_3OH formation rate over Ga_2O_3 during methanol synthesis. Reaction conditions: H_2/CO_2= 3 (v/v), 250-350 °C, 3 MPa, 6000 mL/g/h.

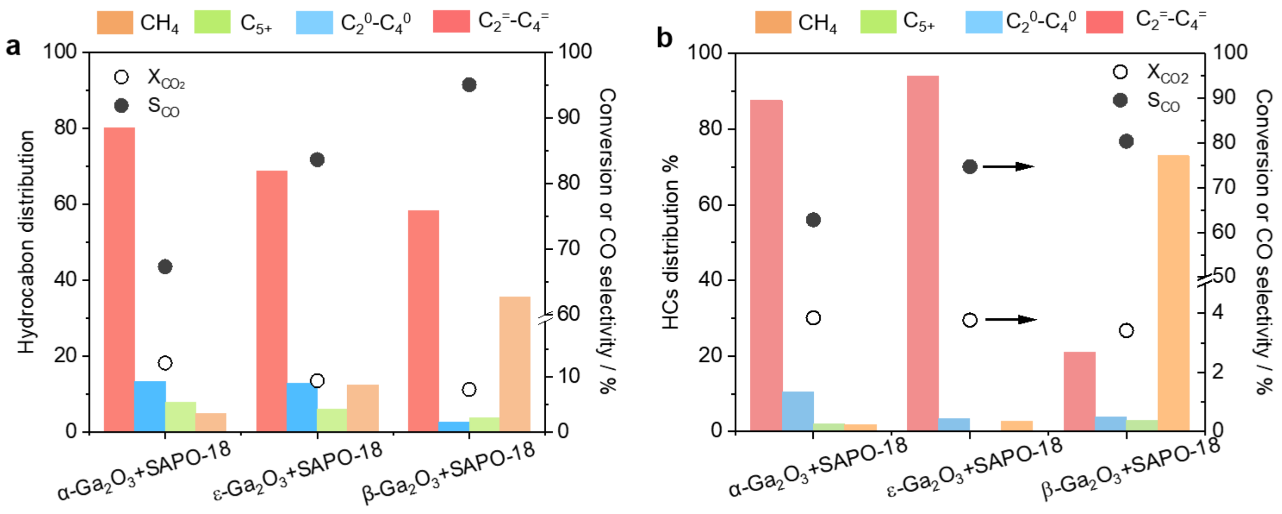

Supplementary Figure 29. **a** Reaction performance of CO_2 hydrogenation over Ga_2O_3-SAPO-18. Reaction conditions: OX/ZEO= 2 (mass ratio, 40-60 mesh), H_2/CO_2= 3 (v/v), 350 °C, 3 MPa, 8000 mL/g/h. **b** Reaction performance of CO_2 hydrogenation over Ga_2O_3-SAPO-18. Reaction conditions: OX/ZEO= 0.5 (mass ratio, 40-60 mesh), H_2/CO_2= 3 (v/v), 350 °C, 3 MPa, GHSV= 20000 mL/g/h.

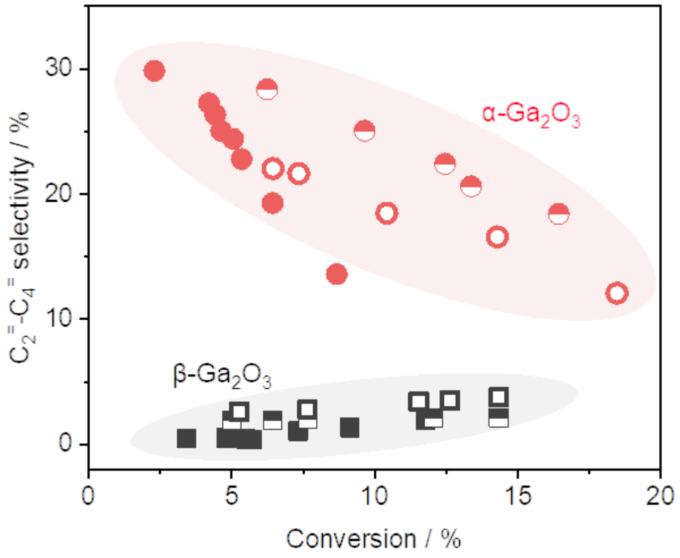

Supplementary Figure 30. $C_2^=$-$C_4^=$ selectivity as a function of CO_2 conversion of different Ga_2O_3-SAPO-18 samples under different space velocity (2000-16000 mL/g/h). α-Ga_2O_3: SAPO-18 = 0.5 (Red solid circle), 2 (Red half-filled circle), 4 (Red hollow circle). β-Ga_2O_3: SAPO-18 = 0.5 (Black solid square), 2 (Black half-filled square), 4 (Black hollow square).

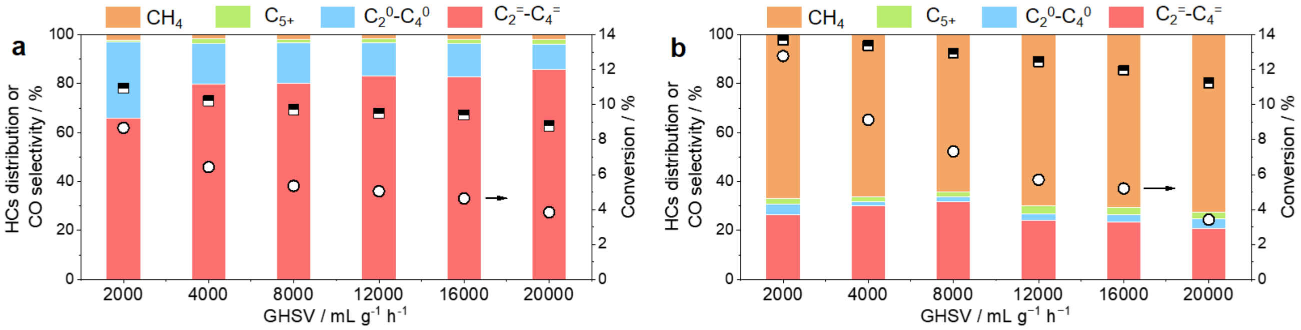

Supplementary Figure 31. Reaction performance of CO_2 hydrogenation over Ga_2O_3-SAPO-18 under different gas hourly space velocity. α-Ga_2O_3-SAPO-18 (a), β-Ga_2O_3-SAPO-18 (b). Reaction conditions: OX/ZEO= 0.5 (mass ratio, 40-60 mesh), H_2/CO_2= 3 (v/v), 350 °C, 3 MPa.

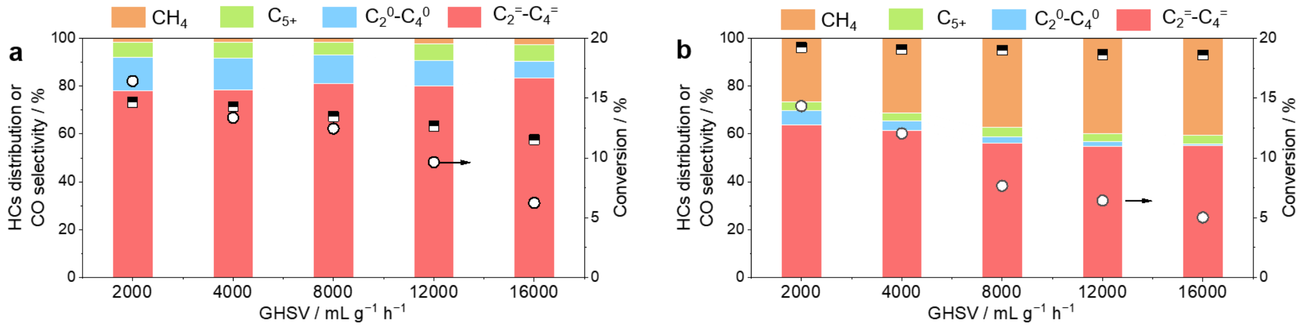

Supplementary Figure 32. Reaction performance of CO_2 hydrogenation over Ga_2O_3-SAPO-18 under different gas hourly space velocity. α-Ga_2O_3-SAPO-18 (a), β-Ga_2O_3-SAPO-18 (b). Reaction conditions: OX/ZEO= 2 (mass ratio, 40-60 mesh), H_2/CO_2= 3 (v/v), 350 °C, 3 MPa.

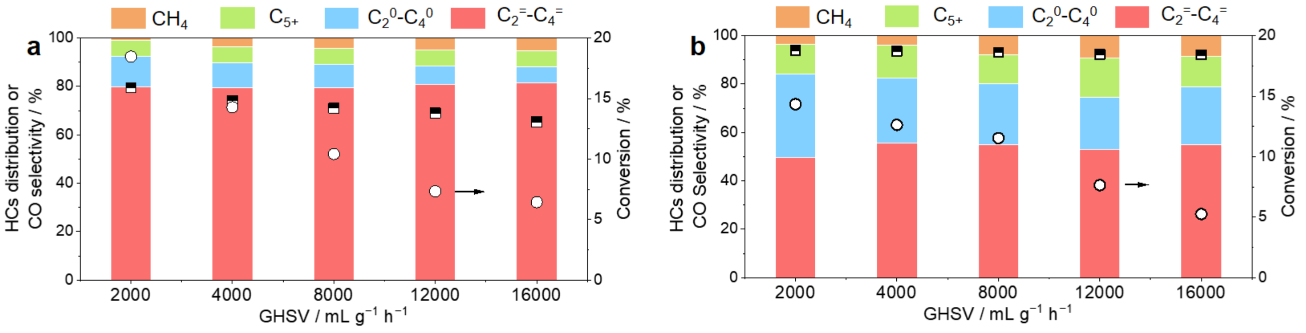

Supplementary Figure 33. Reaction performance of CO_2 hydrogenation over Ga_2O_3-SAPO-18 under different gas hourly space velocity. α-Ga_2O_3-SAPO-18 (a), β-Ga_2O_3-SAPO-18 (b). Reaction conditions: OX/ZEO= 4 (mass ratio, 40-60 mesh), H_2/CO_2= 3 (v/v), 350 °C, 3 MPa.

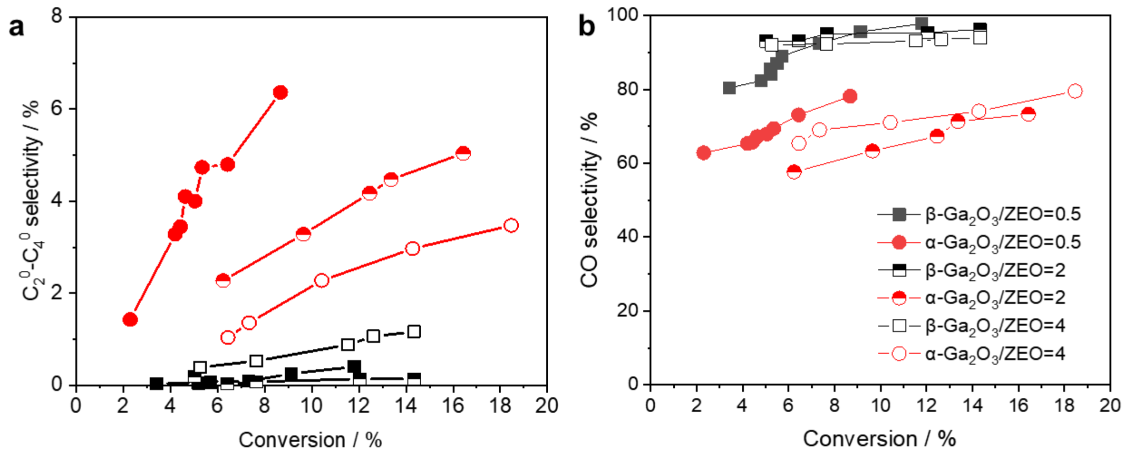

Supplementary Figure 34. a C_2^0-C_4^0 selectivity as a function of CO_2 conversion of different Ga_2O_3-SAPO-18 samples under different space velocity. **b** CO selectivity as a function of CO_2 conversion of different Ga_2O_3-SAPO-18 samples under different space velocity. Reaction conditions: H_2/CO_2= 3 (v/v), 350 °C, 3 MPa.

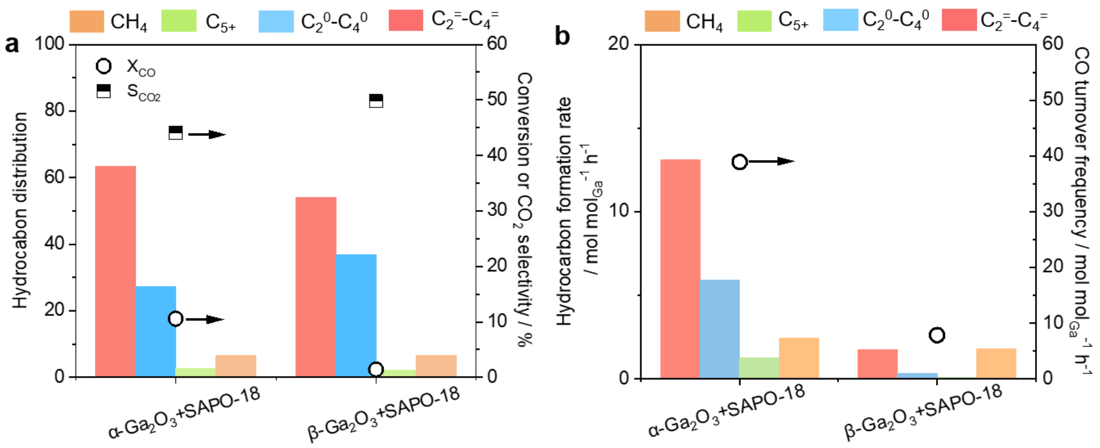

Supplementary Figure 35. a Hydrocarbon distribution, CO_2 selectivity and CO conversion over Ga_2O_3-SAPO-18. Reaction conditions: OX/ZEO= 2 (mass ratio, 40-60 mesh), H_2/CO= 2.5 (v/v), 350 °C, 2.5 MPa, 4000 mL/g/h. **b** Hydrocarbon formation rate and CO turnover frequency normalized by the surface exposed Ga^{3+} atoms. Reaction conditions: OX/ZEO= 2 (mass ratio, 40-60 mesh), H_2/CO_2= 2.5 (v/v), 350 °C, 2.5 MPa, 10000 mL/g/h.

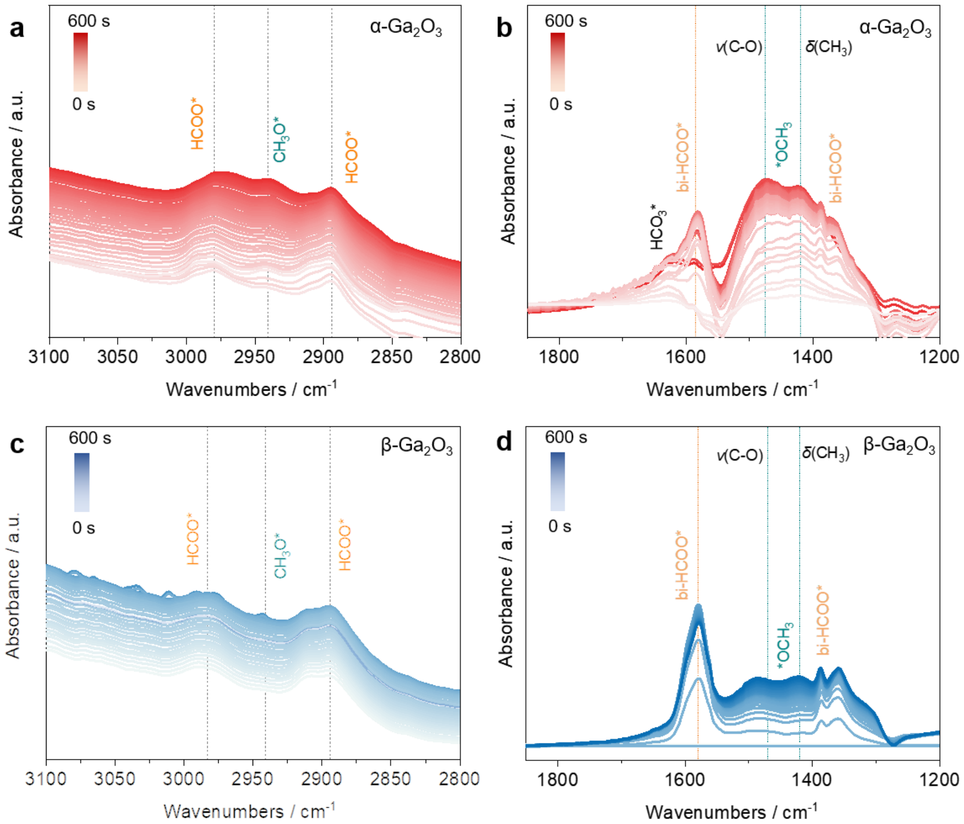

Supplementary Figure 36. *In-situ* FTIR spectra of Ga_2O_3 samples. **a, b** *In-situ* FTIR spectra of surface species under H_2 and subsequently switched to CO_2 over α-Ga_2O_3. **c, d** *In-situ* FTIR spectra of surface species under H_2 and subsequently switched to CO_2 over β-Ga_2O_3. Reaction conditions: 1 MPa, 350 °C, 10 ml/min CO_2 or 30 ml/min H_2.

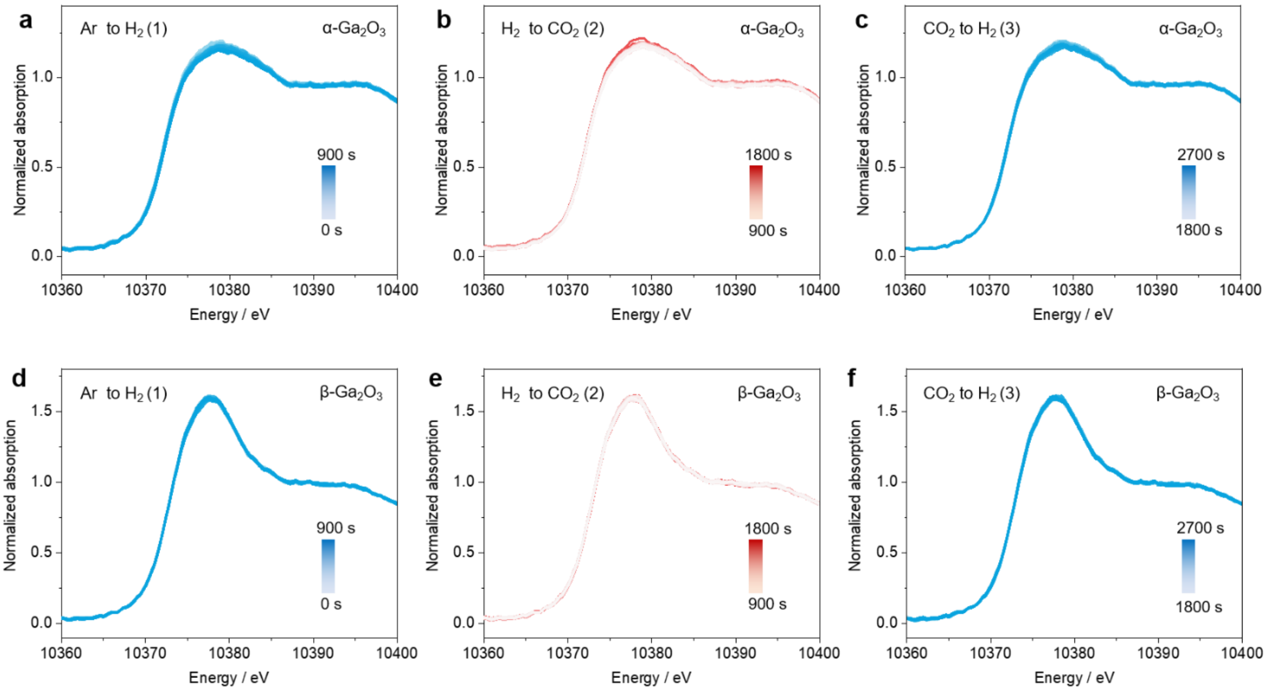

Supplementary Figure 37. *In-situ* time-resolved XANES for α-Ga_2O_3 (a-c) and β-Ga_2O_3 (d-f) under H_2 and CO_2. Reaction conditions: 350 °C, 1 MPa.

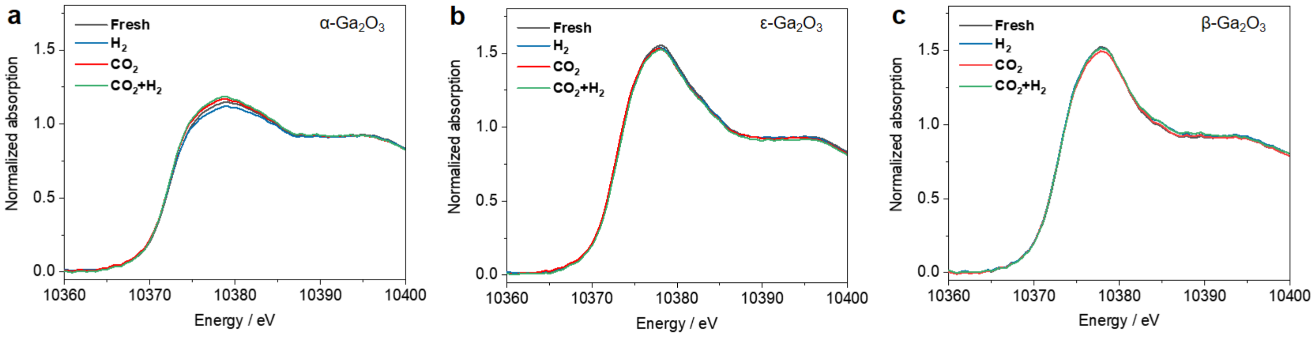

Supplementary Figure 38. *In-situ* XANES spectra in the near edge region of the Ga K-edge for α-Ga_2O_3 (a), ε-Ga_2O_3 (b) and β-Ga_2O_3 (c). The black, blue, red and green colors refer to treatment conditions of Ar, H_2, CO_2 and H_2+CO_2 (H_2/CO_2= 3 (v/v)), respectively. Reaction conditions: 350 °C, 1 MPa.

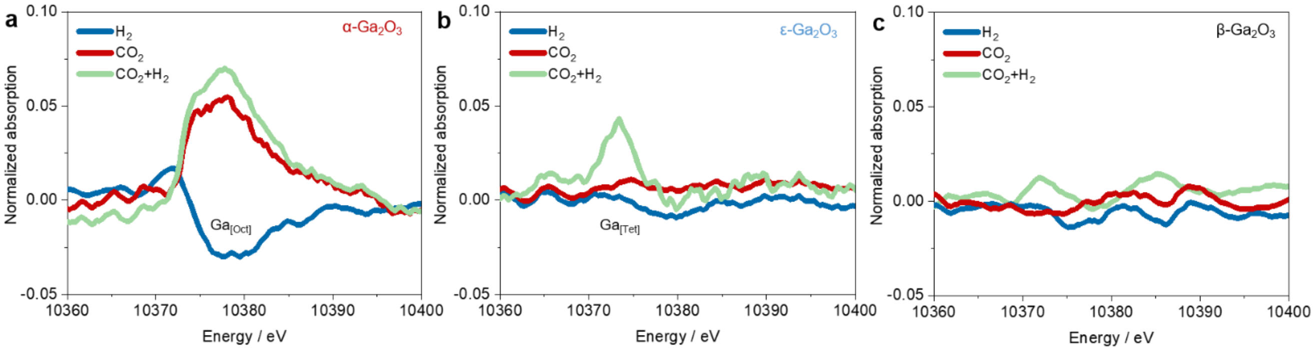

Supplementary Figure 39. Subtraction results of *in-situ* XANES spectra in the near edge region of the Ga K-edge for α-Ga_2O_3 (a), ε-Ga_2O_3 (b) and β-Ga_2O_3 (c). The blue color refer to treatment conditions of H_2 subtracting Ar-treatment spectra as background. The red and green colors refer to treatment conditions of CO_2 and H_2+CO_2 (H_2/CO_2= 3 (v/v)) subtracting H_2-treatment spectra as background respectively. Reaction conditions: 350 °C, 1 MPa.

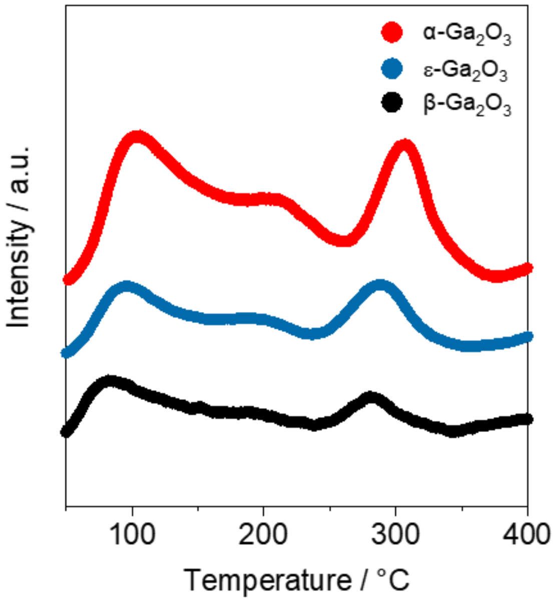

Supplementary Figure 40. CO_2-TPD profiles of different Ga_2O_3 samples normalized by specific surface area.

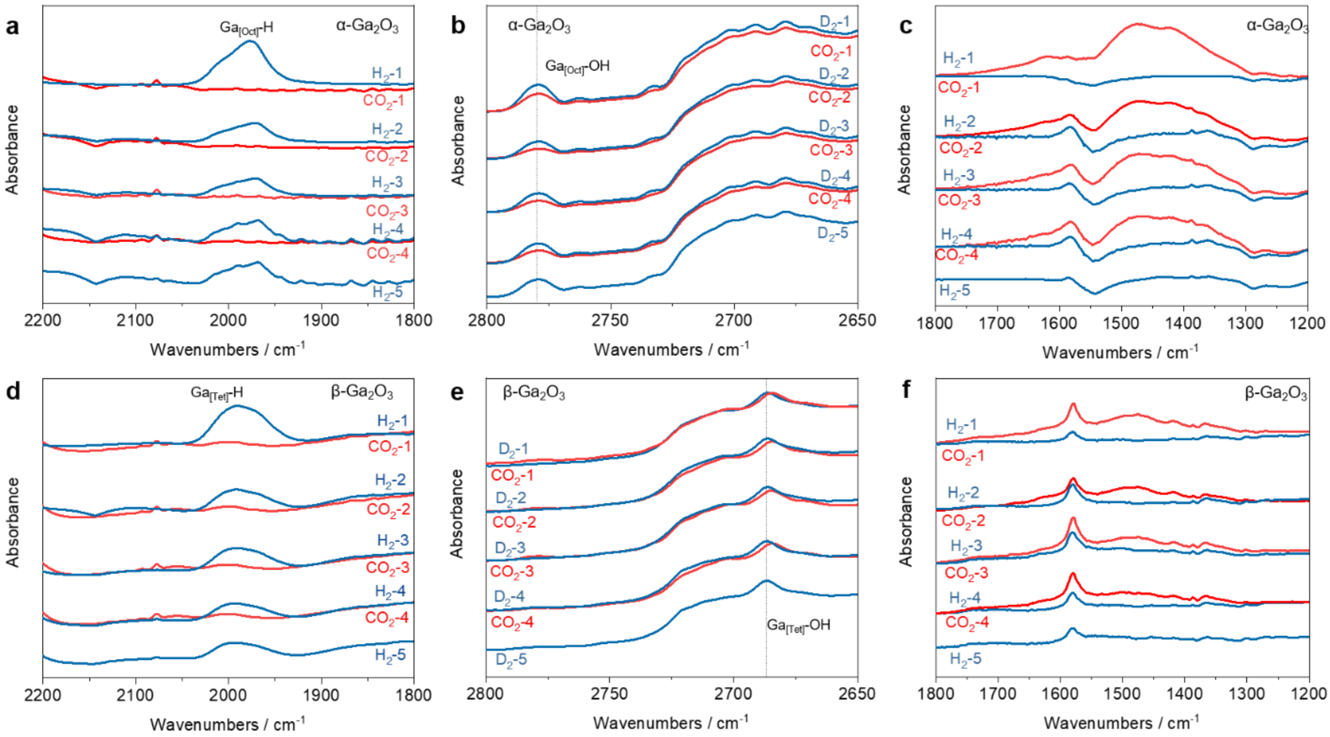

Supplementary Figure 41. *In-situ* FTIR spectra of surface species in H_2/D_2-CO_2 switching experiments from H_2/D_2 (Blue) to CO_2 (Red) and back to H_2. α-Ga_2O_3 (a-c), β-Ga_2O_3 (d-f). Reaction conditions: 1 MPa, 350 °C, 10 ml/min CO_2 and 30 ml/min H_2 (D_2).

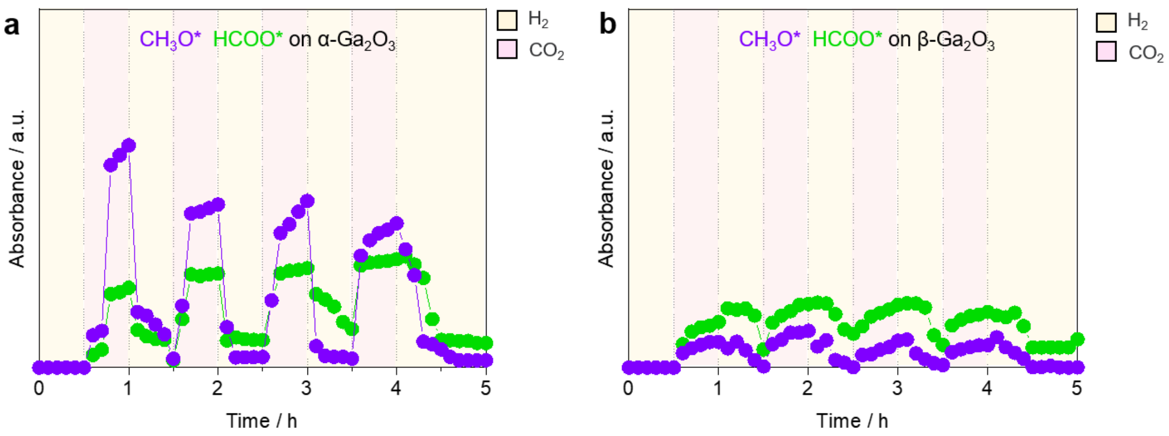

Supplementary Figure 42. H_2-CO_2 switching experiments from H_2 (Light yellow) to CO_2 (Light red) and back to H_2 at 350 °C and 1 MPa. **a** The IR peak intensity of CH_3O^* and $HCOO^*$ over α-Ga_2O_3. **b** The IR peak intensity of CH_3O^* and $HCOO^*$ over β-Ga_2O_3.

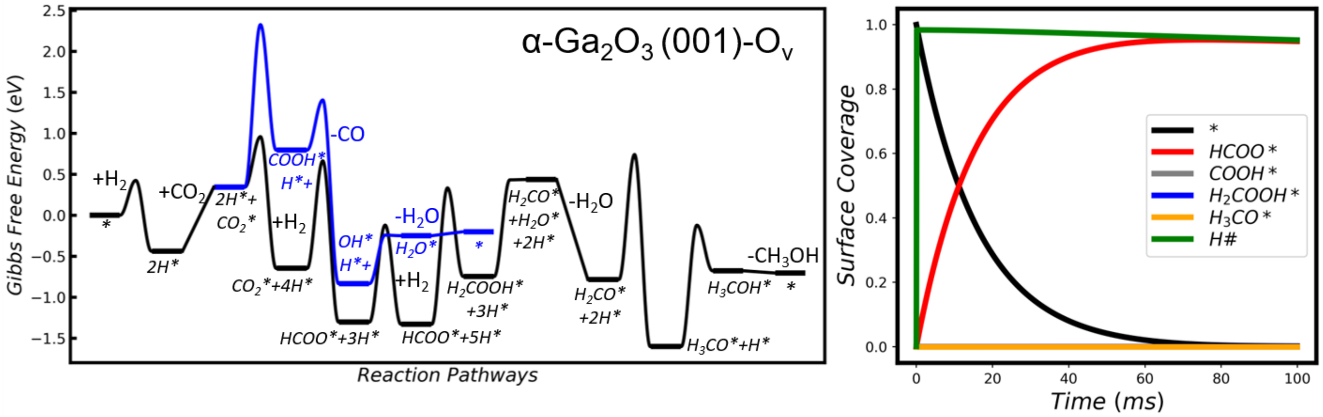

Supplementary Figure 43. Reaction diagram of CO_2 hydrogenation on oxygen vacancy on the (001) surface of the α-Ga_2O_3 model.

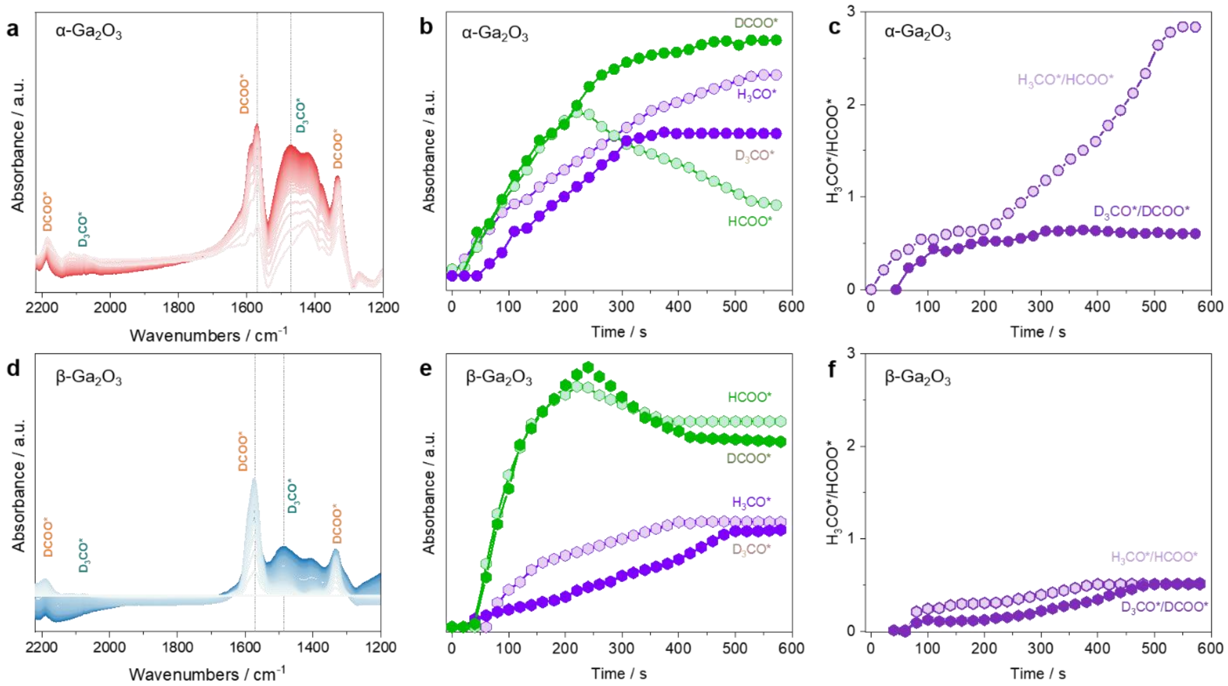

Supplementary Figure 44. *In-situ* FTIR spectra of Ga_2O_3 samples. **a** *In-situ* FTIR spectra of surface species under D_2 and subsequently switched to CO_2 over α-Ga_2O_3. **b** The time-depednent IR peak intensity of CH_3O*, HCOO*, CD_3O* and DCOO*, over α-Ga_2O_3. **c** The time-depednent CH_3O*/HCOO* ratio and CD_3O*/DCOO* ratio over α-Ga_2O_3. **d** *In-situ* FTIR spectra of surface species under D_2 and subsequently switched to CO_2 over β-Ga_2O_3. **e** The time-depednent IR peak intensity of CH_3O*, HCOO*, CD_3O* and DCOO*, over β-Ga_2O_3. **f** The time-depednent CH_3O*/HCOO* ratio and CD_3O*/DCOO* ratio over β-Ga_2O_3. Reaction conditions: 1 MPa, 350 °C, 10 ml/min CO_2 or 30 ml/min D_2.

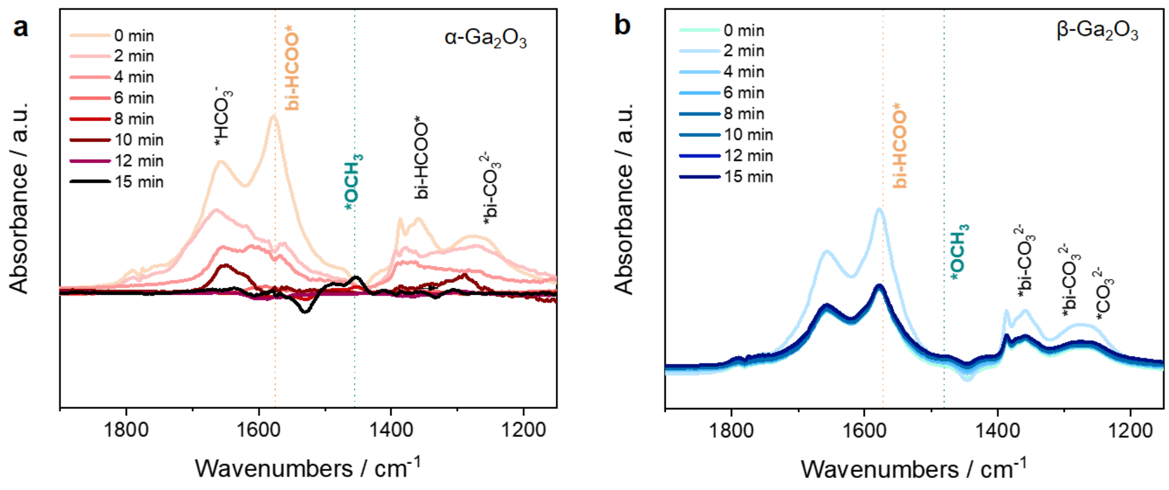

Supplementary Figure 45. *In-situ* FTIR spectra of Ga_2O_3 samples. **a** *In-situ* FTIR spectra of surface species for formate hydrogenation over α-Ga_2O_3. **b** *In-situ* FTIR spectra of surface species for formate hydrogenation over β-Ga_2O_3. Reaction conditions: 350 °C, 10 ml/min N_2 with formate.

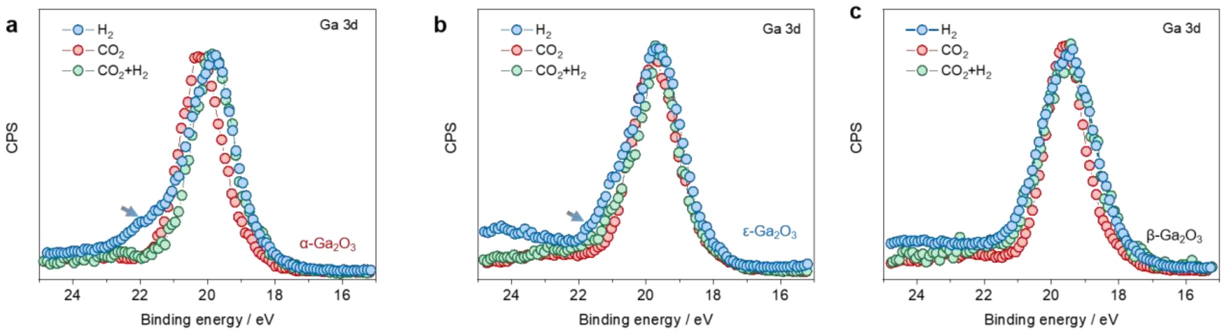

Supplementary Figure 46. *In-situ* XPS spectra of Ga 3*d* for α-Ga_2O_3 (a), ε-Ga_2O_3 (b) and β-Ga_2O_3 (c). The blue, red and green colors refer to treatment conditions of H_2, CO_2 and H_2+CO_2 (H_2/CO_2= 3 (v/v)), respectively. Reaction conditions: 350 °C, 3 MPa.

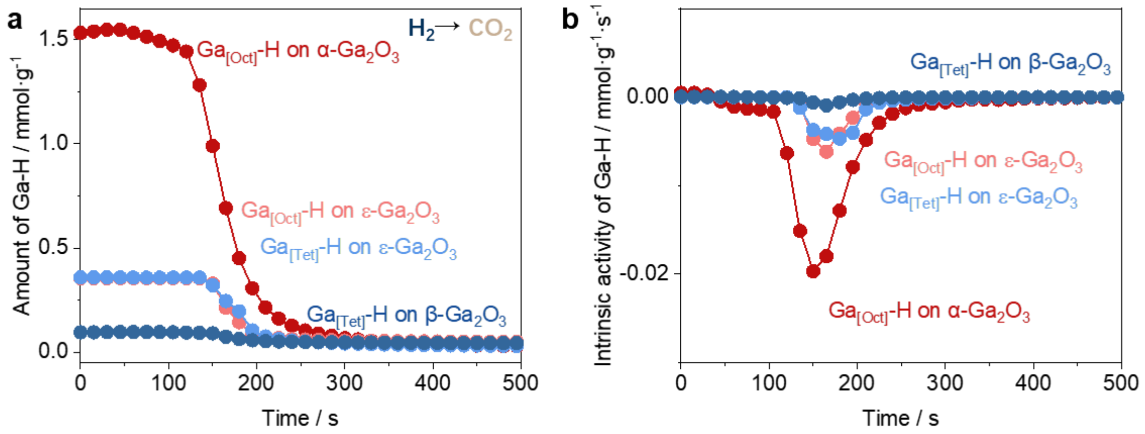

Supplementary Figure 47. a The amount of surface Ga-H contacting CO_2 versus time at 350°C over different Ga_2O_3 phase. **b** Conversion rate of Ga-H reacted with CO_2 versus time.

Note: In *in situ* FTIR, the intensity of Ga-H peak declined with the CO_2 inlet into the transmission cell. Meanwhile, the peak of HCOO* and CH_3O* species appeared and increased with CO_2 inlet. Differential processing was performed on the amounts of Ga-H species as a function of CO_2 exposure time to obtain the conversion rate of hydride in three different phases. It is found that conversion rate of the hydrides on octahedra Ga^{3+} of α-Ga_2O_3 is two times higher than hydrides on octahedra Ga^{3+} of the ε phase and is much higher than tetrahedral Ga^{3+} of β-Ga_2O_3 (Supplementary Figure S47).

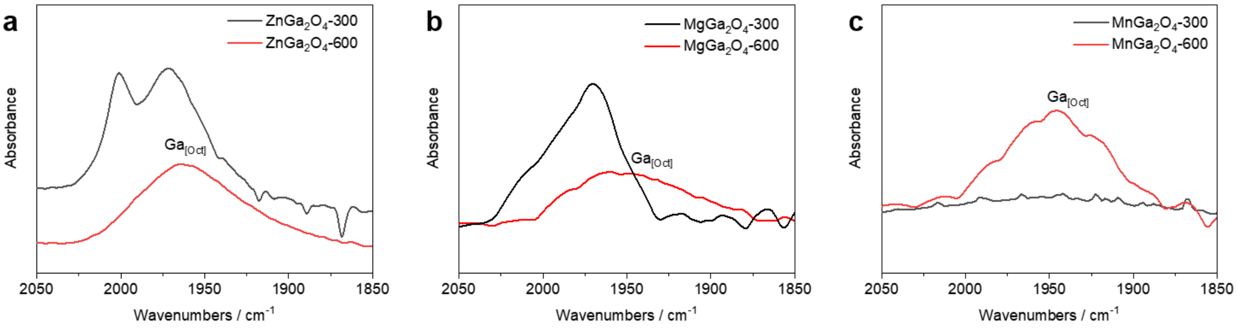

Supplementary Figure 48. H_2-IR for Ga-based spinel samples contacting with H_2 at 350 °C, 1 MPa. $ZnGa_2O_4$ (a), $MgGa_2O_4$ (b) and $MnGa_2O_4$ (c).

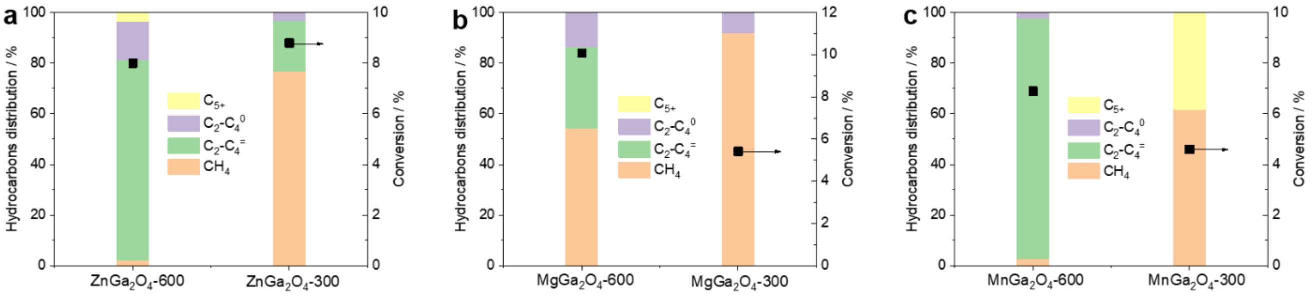

Supplementary Figure 49. Reaction performance of CO_2 hydrogenation over Ga-based spinel-SAPO-18. $ZnGa_2O_4$ (a), $MgGa_2O_4$ (b) and $MnGa_2O_4$ (c). Reaction conditions: OX/ZEO= 2 (mass ratio, 20-40 mesh), H_2/CO_2= 3 (v/v), 350 °C, 3 MPa, 8000 mL g^{-1} h^{-1}.

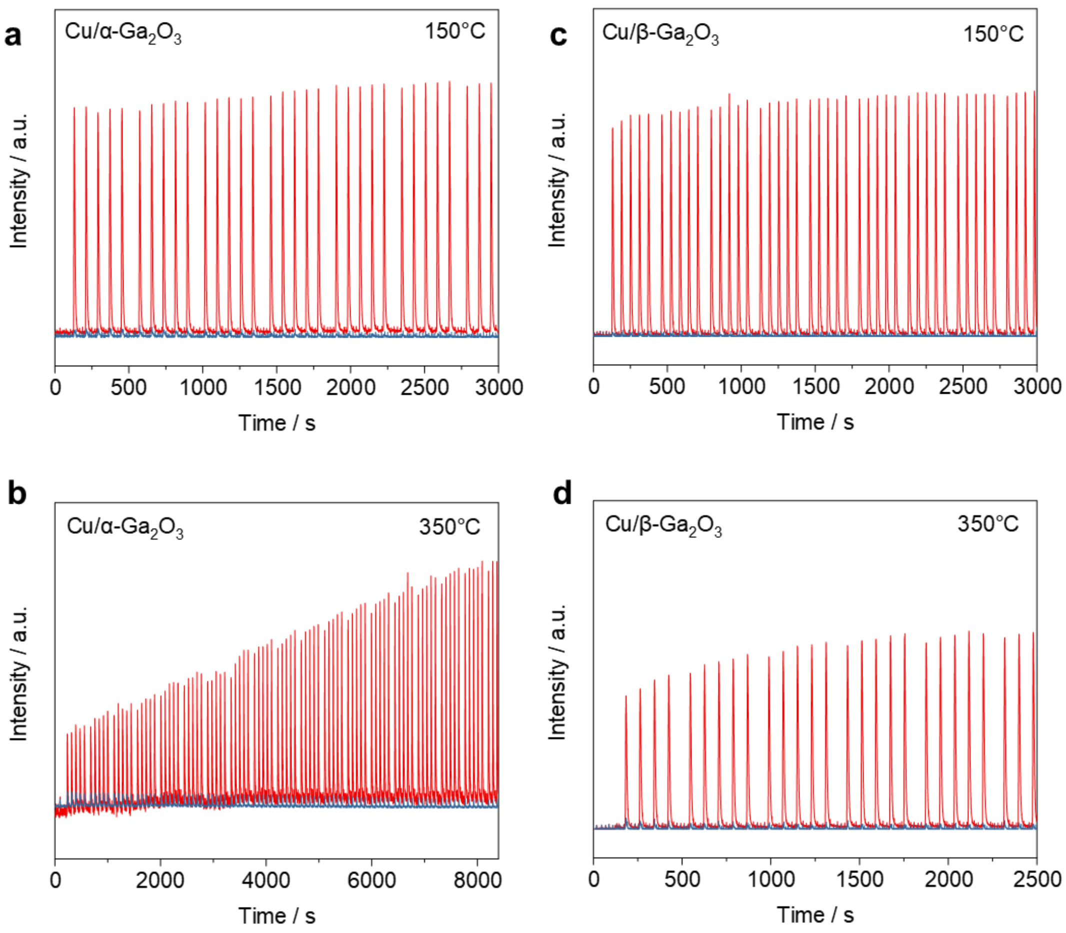

Supplementary Figure 50. a, c Hydrogen consumption with *m/z*=2 (H_2, red), *m/z*=3 (HD, blue) of Cu/Ga_2O_3 during H_2-exchange experiment at 150 °C after the catalysts were saturated with D_2 at 350 °C. **b, d** Hydrogen consumption with *m/z*=2 (H_2, red), *m/z*=3 (HD, blue) of Cu/Ga_2O_3 during H_2-exchange experiment at 350 °C after the catalysts were saturated with D_2 at 350 °C.

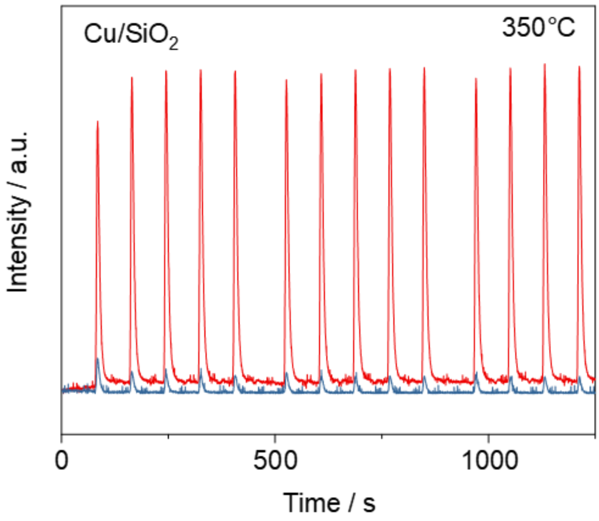

Supplementary Figure 51. Hydrogen consumption with *m*/*z*=2 (H_2, red), *m*/*z*=3 (HD, blue) of Cu/SiO_2 during H_2-exchange experiment at 350 °C after the catalysts were saturated with D_2 at 350 °C.

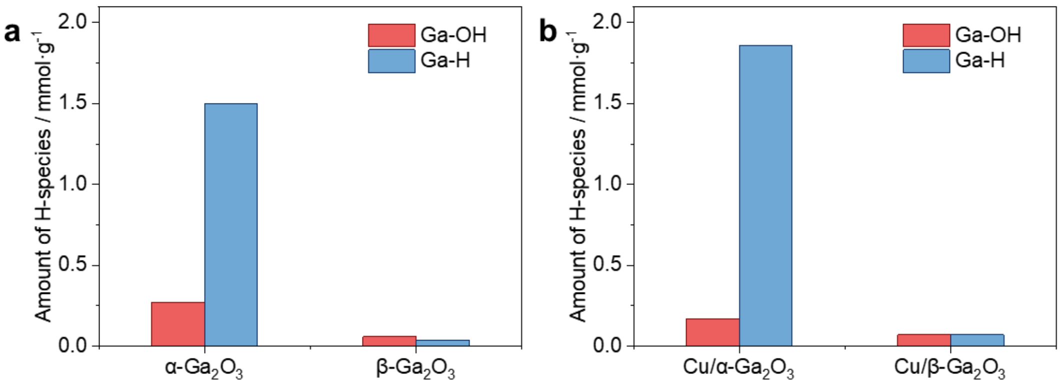

Supplementary Figure 52. a Amount of surface hydrogen species over Ga_2O_3. **b** Amount of surface hydrogen species over Cu/Ga_2O_3.

Note: We also conducted TKA-MS experiments to quantify surface hydrogen species over Cu/Ga_2O_3 (Supplementary Fig. 50). Using inert SiO_2 loaded with the same amount of Cu as a reference catalyst, we found that there are relatively fewer metal-hydrogen bonds on Cu/SiO_2 (Supplementary Fig. 51). After deducting Cu-H, the amount of Ga-H species on Cu/Ga_2O_3 were obtained as shown in Supplementary Fig. 52. The Ga-H on Cu/α-Ga_2O_3 was measured to be higher than -OH. Thus, homolytic H_2 dissociation also occurs on Cu/α-Ga_2O_3 to produce high-density of Ga-H, which is similar with α-Ga_2O_3 (Supplementary Fig. 52).

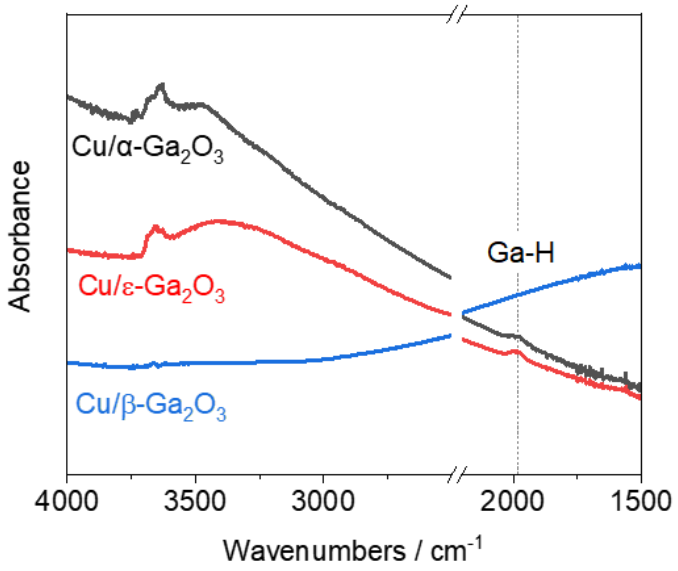

Supplementary Figure 53. *In-situ* FTIR spectra of surface species over Cu/Ga_2O_3 when contacting H_2 at 350°C.

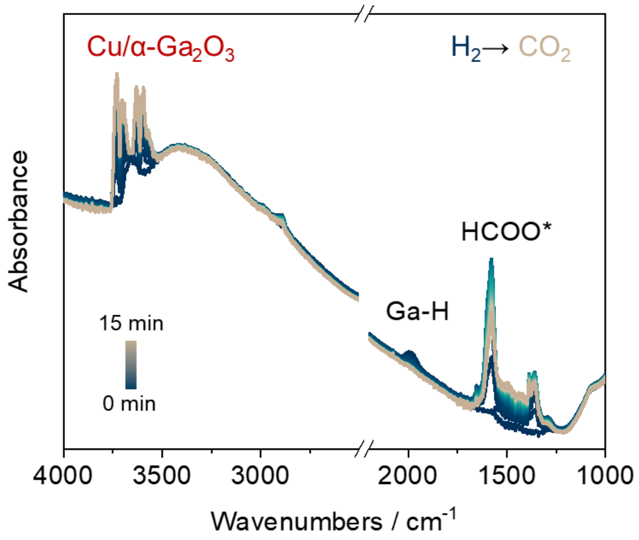

Supplementary Figure 54. *In-situ* FTIR spectra of surface species when switching H_2 to CO_2 over Cu/α-Ga_2O_3 at 350°C.

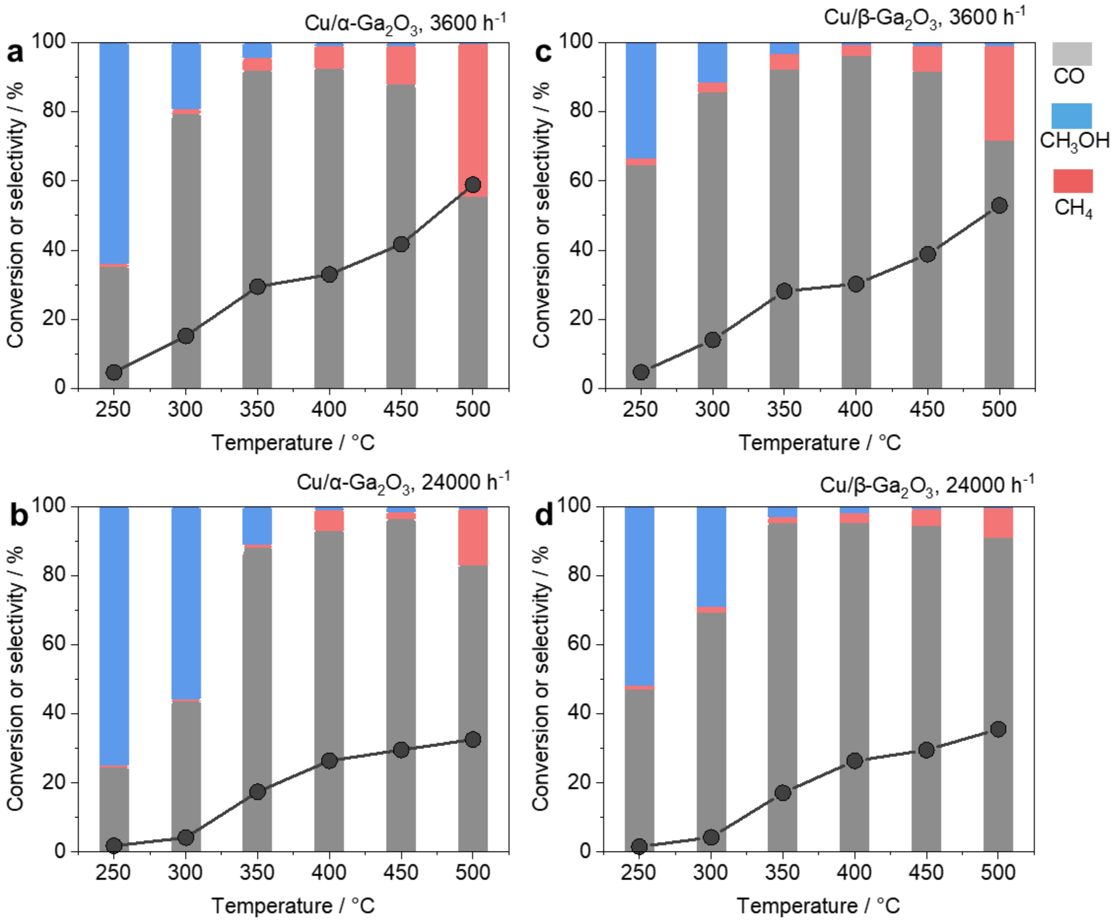

Supplementary Figure 55. Catalytic performance of CO_2 hydrogenation over Cu/Ga_2O_3. Reaction conditions: H_2/CO_2= 3 (v/v), 250-500 °C, 4 MPa, 3600 ml/g_{cat}/h (a, c), 24000 ml/g_{cat}/h (b, d).

Note: The catalytic test for CO_2 hydrogenation to methanol and CO is conducted at 250-500°C and 4 MPa. The test at high GHSV (24000 ml/g_{cat}/h) is also carried out to obtain the intrinsic activity of Cu/Ga_2O_3. Methanol is produced mainly under low-temperature condition (250-350°C) and CO is produced under high-temperature condition (350-500°C) (Supplementary Fig. 55).

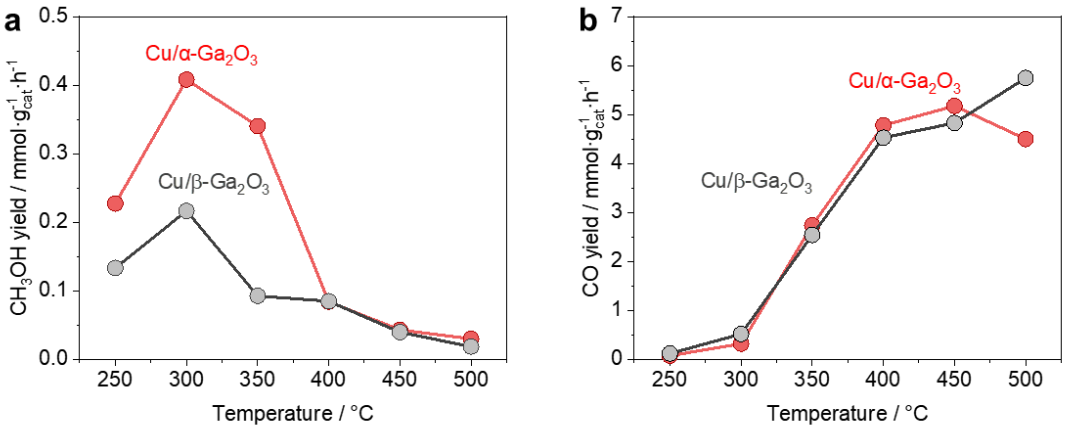

Supplementary Figure 56. a Yield of methanol in CO_2 hydrogenation over Cu/Ga_2O_3. **b** Yield of CO in CO_2 hydrogenation over Cu/Ga_2O_3.Reaction conditions: H_2/CO_2= 3 (v/v), 250-500 °C, 4 MPa, 24000 ml/g_{cat}/h.

Supplementary Tables

Supplementary Table 1. Structural properties and number of activated hydrogen species on Ga_2O_3 surface.

Catalysts	Size [a] (nm)	BET area [b] (m^2/g)	$H_{[surface]}$ [c] (mmol/g)	$H_{[surface]}$ [c] (nm^{-2})	$OH_{[surface]}$ [c] (mmol/g)	$OH_{[surface]}$ [c] (nm^{-2})	H/OH (mol/mol)
α-Ga_2O_3	10	81.5	1.50	11.2	0.27	2.0	5.6
ε-Ga_2O_3	9	94.8	0.72	4.7	0.55	3.6	1.3
β-Ga_2O_3	45	47.8	0.04	0.5	0.06	0.8	0.7

[a] calculated by Scherrer equation based on XRD results. [b] obtained from N_2 physisorption. [c] determined by H_2-exchange experiment.

Supplementary Table 2. The physical property of Ga_2O_3.

Catalysts	Proportions of octahedral Ga^{3+} [a]	Proportions of surface octahedral Ga^{3+} [b]	Proportions of surface tetrahedral Ga^{3+} [b]
α-Ga_2O_3	99%	88%	12%
ε-Ga_2O_3	85%	46%	54%
β-Ga_2O_3	50%	17%	83%

[a] Determined via XANES. [b] Determined via H_2-IR.

Supplementary Table 3. The surface energy and Wulff area ratio of three Ga_2O_3 crystals.

No.	Crystal	Surface	Surface energy (J/m^2)	Wulff Area ratio (%)
1	α-Ga_2O_3	s_001	1.11	37
2	α-Ga_2O_3	s_110	1.43	38
3	α-Ga_2O_3	s_101	1.46	0
4	α-Ga_2O_3	s_010	1.53	8
5	α-Ga_2O_3	s_100	1.53	0
6	α-Ga_2O_3	s_011	1.61	17
7	α-Ga_2O_3	s_111	1.68	0
8	β-Ga_2O_3	s_100	0.48	55
9	β-Ga_2O_3	s_001	1.15	14
10	β-Ga_2O_3	s_101	1.28	4
11	β-Ga_2O_3	s_111	1.29	27
12	β-Ga_2O_3	s_011	1.45	0
13	β-Ga_2O_3	s_010	1.50	0
14	β-Ga_2O_3	s_110	1.68	0
15	ε-Ga_2O_3	s_011	0.86	66
16	ε-Ga_2O_3	s_001	1.23	1
17	ε-Ga_2O_3	s_101	1.31	19
18	ε-Ga_2O_3	s_110	1.38	11
19	ε-Ga_2O_3	s_111	1.39	3
20	ε-Ga_2O_3	s_100	1.47	0
21	ε-Ga_2O_3	s_010	1.49	0

Supplementary Table 4. Ga atomic density on different crystal planes of Ga_2O_3.

Crystal plane	Ga atomic density (atoms nm^{-2})		
	α-Ga_2O_3	ε- Ga_2O_3	β-Ga_2O_3
[100]	11.95	5.92	5.67
[010]	11.95	5.92	5.63
[001]	1.73	5.92	13.08
[110]	11.95	10.47	2.73
[101]	1.56	10.47	2.23
[011]	1.56	10.47	5.05
[111]	8.48	11.96	12.16
Average	7.02	8.73	6.65

Supplementary Table 5. Unit cell parameters of Ga_2O_3.

Unit cell parameters	α-Ga_2O_3	ε-Ga_2O_3	β-Ga_2O_3
a (Å)	4.9825	8.22	12.227
b (Å)	4.9825	8.22	3.0389
c (Å)	13.433	8.22	5.8079
α (°)	90.00	90.00	90.00
β (°)	90.00	90.00	103.82
γ (°)	120.00	90.00	90.00
V (nm^3)	0.29	0.56	0.21
Z	6.00	10.67	4.00
M (g mol^{-1})	187.44	187.44	187.44

Supplementary Table 6. Number of Ga cation exposing on Ga_2O_3 surface.

Catalysts	$Ga_{[oct, surface]}/Ga_{[tet, surface]}$ [a] (mol/mol)	$Ga_{[surface]}$ [b] (mmol/g)	$Ga_{[surface]}$ [b] (nm^{-2})	$Ga_{[oct, surface]}$ [b] (mmol/g)	$Ga_{[tet, surface]}$ [b] (mmol/g)
α-Ga_2O_3	7.3	0.94	7.0	0.83	0.11
ε-Ga_2O_3	0.9	1.08	7.0	0.50	0.58
β-Ga_2O_3	0.2	0.53	6.7	0.09	0.44

[a] determined by H_2-IR. [b] determined based on the surface areas and average Ga^{3+} densities for the α-, ε-, β-phases.

Supplementary Table 7. Coverage of activated hydrogen species on Ga_2O_3 surface.

Catalysts	$H_{[surface]}$/ $Ga_{[surface]}$ (mol/mol)	$OH_{[surface]}$/ $Ga_{[surface]}$ (mol/mol)	$H_{[surface]}/O_{[surface]}$ (mol/mol)	Total H (H^-+OH) density (mmol/g)	Total surface atom (Ga+O) density (mmol/g)
α-Ga_2O_3	1.60	0.29	0.24	1.77	2.05
ε-Ga_2O_3	0.67	0.51	0.38	1.27	2.54
β-Ga_2O_3	0.08	0.11	0.08	0.10	1.30

Supplementary Table 8. The valence state analysis of different Ga atoms for three Ga_2O_3 crystals based on Bader charge.

Crystal	Environment	Ga coordination	Ga valence state (e^-)
α-Ga_2O_3	bulk	$Ga_{6c[oct]}$	+1.7
	(001)	Ga_{6c}	+1.6
	(001)-O_v	Ga_{2c}	+1.0
	(001)-O_v-H	Ga_{2c}	+1.3
ε-Ga_2O_3	Bulk	$Ga_{6c[oct]}$	+1.7
	Bulk	$Ga_{4c[tet]}$	+1.7
	(011)	Ga_{4c}	+1.6
	(011)-O_v	Ga_{3c}	+1.0
	(011)-O_v-H	Ga_{3c}	+1.4
β-Ga_2O_3	Bulk	$Ga_{6c[oct]}$	+1.8
	Bulk	$Ga_{4c[tet]}$	+1.7
	(100)	Ga_{5c}	+1.7

Supplementary Table 9. The Bader charge and core-level chemical shift analysis on α-Ga_2O_3 (001) surface with different surface environments.

Surface environment	Bader charge	Binding energy of Ga 3d in XPS (eV)	Core-level chemical shift (eV)
Perfect α-Ga_2O_3 (001)	1.39 e^-	27.052	0
O-defective α-Ga_2O_3 (001)	1.41 e^-	27.415	0.363
O-defective α-Ga_2O_3 (001) with the presence of OH groups	2.21 e^-	27.487	0.435
O-defective α-Ga_2O_3 (001) with the presence of OH groups and GaH species	1.66 e^-	27.611	0.559

Supplementary Table 10. The transition states (TS) analysis.

Initial State	Final State	TS imaginary frequency (cm^{-1})
CO_2*+4H*	HCOO*+3H*	191.0
HCOO*+5H*	H_2COOH*+3H*	304.7
H_2COOH*+3H*	H_2CO*+H_2O*+2H*	846.8
CH_2O*+2H*	CH_3O*+H*	1241
CH_3O*+H*	CH_3OH*	1120.9
CO_2*+2H*	COOH*+H*	101.9
COOH*+H*	OH*+H*+CO	280.1
OH*+H*	H_2O	89.6

Supplementary Table 11. The information of reagents used in method section.

Chemicals	Commercial sources	Purity
Gallium nitrate hydrate ($Ga(NO_3)_2{\cdot}xH_2O$)	Aladdin	AR, 99.9%
Liquid ammonia solution ($NH_3{\cdot}H_2O$)	Sinopharm	25-28%
Anhydrous ethanol (C_2H_5OH)	Sinopharm	AR
Hydrazine monohydrate ($N_2H_4{\cdot}H_2O$)	Sinopharm	98%
Aluminum hydroxide hydrate ($Al(OH)_3{\cdot}6H_2O$)	Aladdin	55 wt% Al_2O_3
phosphoric acid (H_3PO_4)	Aladdin	85wt% in H_2O
Silica sol	Aladdin	30wt% SiO_2
N, N-diisopropylethylamine ($C_8H_{19}N$)	Aladdin	99%
Zinc nitrate hexahydrate ($Zn(NO_3)_2{\cdot}6H_2O$)	Aladdin	99.9%
Magnesium nitrate hexahydrate ($Mg(NO_3)_2{\cdot}6H_2O$)	Aladdin	99.9%
50 wt% aqueous solutions of $Mn(NO_3)_2$	Aladdin	50%
Sodium hydroxide (NaOH)	Sinopharm	97%
Sodium bicarbonate ($NaHCO_3$)	Sinopharm	AR
Hydrated copper nitrate ($Cu(NO_3)_2{\cdot}xH_2O$)	Aladdin	AR
Silicon dioxide (SiO_2)	Aladdin	5-20 nm, 99.5%

Supplementary References

1. Li, L., Wei, W. & Behrens, M. Synthesis and characterization of α-, β-, and γ-Ga_2O_3 prepared from aqueous solutions by controlled precipitation. *Solid State Sci.* **14**, 971-981 (2012).
2. Sharma, A. *et al.* Nano-structured phases of gallium oxide (GaOOH, α-Ga_2O_3, β-Ga_2O_3, γ-Ga_2O_3, δ-Ga_2O_3, and ε-Ga_2O_3): fabrication, structural, and electronic structure investigations. *Int. Nano Lett.* **10**, 71-79 (2020).
3. Nishi, K. *et al.* Deconvolution analysis of Ga K-edge XANES for quantification of gallium coordinations in oxide environments. *J. Phys. Chem. B* **102**, 10190-10195 (1998).
4. Akatsuka, M. *et al.* XAFS analysis for quantification of the gallium coordinations in Al_2O_3-supported Ga_2O_3 photocatalysts. *J. Phys: Conf. Ser.* **712** (2016).
