# Supplementary material for: Homolytic H2 dissociation for enhanced hydrogenation catalysis on oxides
Source: Nat Commun. 2024 Jan 15;15:540. doi: 10.1038/s41467-024-44711-7 (PMC10789776; doi:10.1038/s41467-024-44711-7)
Supplement: Supplementary file 3 — Description of Additional Supplementary Files [file 41467_2024_44711_MOESM3_ESM.pdf]

Description of Additional Supplementary Files

File Name: Supplementary Data 1
Description: All the bulk, surface and reaction structures of Ga_2O_3.
